# Proteomic analysis across Healthy–NAT–Tumor tissues uncovers clinically relevant biological events in esophageal squamous cell carcinoma

**DOI:** 10.1093/bib/bbag186

**Published:** 2026-04-24

**Authors:** Wei Liu, Wei Wang, Dan-Wei Zheng, Ying-Qin Ran, Ming-Xiao Feng, Dan-Xia Deng, Xiu-E Xu, Li-Yan Xu, Hai-Hua Huang, En-Min Li

**Affiliations:** College of Science, Heilongjiang Institute of Technology, No. 999 Hongqi Street, Daowai District, Harbin 150050, Heilongjiang, China; College of Science, Heilongjiang Institute of Technology, No. 999 Hongqi Street, Daowai District, Harbin 150050, Heilongjiang, China; The Key Laboratory of Molecular Biology for High Cancer Incidence Coastal Chaoshan Area, Institute of Oncologic Pathology, Department of Pathology, Second Affiliated Hospital, Shantou University Medical College, 69 Dongxia North Road, Jinping District, Shantou 515051, Guangdong, China; The Key Laboratory of Molecular Biology for High Cancer Incidence Coastal Chaoshan Area, Institute of Oncologic Pathology, Department of Pathology, Second Affiliated Hospital, Shantou University Medical College, 69 Dongxia North Road, Jinping District, Shantou 515051, Guangdong, China; The Key Laboratory of Molecular Biology for High Cancer Incidence Coastal Chaoshan Area, Institute of Oncologic Pathology, Department of Pathology, Second Affiliated Hospital, Shantou University Medical College, 69 Dongxia North Road, Jinping District, Shantou 515051, Guangdong, China; The Key Laboratory of Molecular Biology for High Cancer Incidence Coastal Chaoshan Area, Institute of Oncologic Pathology, Department of Pathology, Second Affiliated Hospital, Shantou University Medical College, 69 Dongxia North Road, Jinping District, Shantou 515051, Guangdong, China; The Key Laboratory of Molecular Biology for High Cancer Incidence Coastal Chaoshan Area, Institute of Oncologic Pathology, Department of Pathology, Second Affiliated Hospital, Shantou University Medical College, 69 Dongxia North Road, Jinping District, Shantou 515051, Guangdong, China; The Key Laboratory of Molecular Biology for High Cancer Incidence Coastal Chaoshan Area, Institute of Oncologic Pathology, Department of Pathology, Second Affiliated Hospital, Shantou University Medical College, 69 Dongxia North Road, Jinping District, Shantou 515051, Guangdong, China; The Key Laboratory of Molecular Biology for High Cancer Incidence Coastal Chaoshan Area, Institute of Oncologic Pathology, Department of Pathology, Second Affiliated Hospital, Shantou University Medical College, 69 Dongxia North Road, Jinping District, Shantou 515051, Guangdong, China; The Key Laboratory of Molecular Biology for High Cancer Incidence Coastal Chaoshan Area, Institute of Oncologic Pathology, Department of Pathology, Second Affiliated Hospital, Shantou University Medical College, 69 Dongxia North Road, Jinping District, Shantou 515051, Guangdong, China; Chaoshan Branch of State Key Laboratory for Esophageal Cancer Prevention and Treatment, Department of Biochemistry and Molecular Biology, Shantou University Medical College, No. 22 Xinling Road, Jinping District, Shantou 515041, Guangdong, China; Shantou Central Hospital, Shantou Academy Medical Sciences, No. 114 Waima Road, Jinping District, Shantou 515041, Guangdong, China

**Keywords:** esophageal squamous cell carcinoma, proteomics, NAT, molecular subtypes, biomarkers

## Abstract

Esophageal squamous cell carcinoma (ESCC) is a highly lethal malignancy with limited therapeutic progress and a 5-year survival rate below 20%. Normal adjacent-to-tumor (NAT) tissues, widely used as “normal” controls, are increasingly recognized as molecularly distinct from both tumor and healthy tissues, reflecting early carcinogenic alterations rather than a true normal state. Here, we integrated proteomic data from 20 Healthy, 124 NAT, and 124 Tumor tissues to systematically map protein alterations across the full spectrum of ESCC development. Cross-stage analysis identified eight distinct expression modes, capturing stepwise molecular transitions from Healthy to NAT to Tumor. Notably, NAT tissues exhibited extensive early molecular alterations, characterized by pronounced immune activation—particularly in the complement and coagulation cascades—and broad metabolic reprogramming. We further demonstrated that the NAT proteome itself harbors critical clinical information, defining two proteomic subtypes and four immune subtypes that were strongly associated with patient survival and tumor stage. Based on these features, we developed two prognostic models: (i) an integrated NAT-subtype–pTNM model, which outperformed traditional staging, and (ii) a “US” model, built from proteins consistently upregulated from Healthy to NAT and remaining stable in Tumor samples, which achieved superior predictive performance in the independent test set (5-year AUC = 0.849 for overall survival; 3-year AUC = 0.861 for disease-free survival). Together, these findings extend beyond conventional Tumor–NAT comparisons, offering molecular insights and clinically relevant resources for early detection, patient stratification, and therapeutic development in ESCC.

## Introduction

Esophageal squamous cell carcinoma (ESCC) is the most common form of esophageal cancer, accounting for the majority of ~600 000 new cases and 540 000 deaths worldwide in 2020 [1]. It is particularly prevalent in Eastern Asia and parts of Africa and is characterized by late diagnosis, rapid progression, frequent recurrence, and strong therapy resistance, resulting in a poor 5-year survival rate of only 15%–20% [2, 3]. Although multimodal treatments—including surgery, radiotherapy, chemotherapy, targeted therapy, and immunotherapy—have improved outcomes for some patients, the overall survival benefit remains limited [4]. Comprehensive genomic, transcriptomic, and proteomic studies have provided deeper insights into ESCC pathogenesis, identifying key molecular drivers and potential therapeutic targets [5–9]. However, the clinical translation of these discoveries remains limited, and reliable biomarkers for early detection, molecular classification, and therapeutic targeting are still lacking.

Normal adjacent-to-tumor (NAT) tissues, defined as histologically normal tissues adjacent to tumor lesions, are commonly used as controls in molecular studies. However, because NATs originate from cancer-bearing individuals rather than healthy subjects, they differ from true Healthy tissues. This distinction has drawn increasing attention, and several mechanisms—including regional “field effects” [10, 11], tumor–microenvironmental influences [12], and tumor cell contamination [13]—have been proposed to explain these differences. Recent pan-cancer transcriptomic analyses revealed that NATs represent a molecularly intermediate state between Healthy and Tumor tissues, reflecting early steps of malignant transformation and correlating with patient outcomes [14, 15]. Similar findings have been consistently observed across multiple cancer types, including head and neck, esophageal, gastric, colorectal, and prostate cancers, where NATs show distinct genomic, transcriptomic, and proteomic profiles compared with Healthy counterparts [11, 16–18]. In ESCC, NATs display a somatic mutation frequency ~1.4-fold higher than that of healthy esophageal tissues [19]. Clonal expansion driven by genes such as NOTCH1 and PPM1D has been shown to remodel histologically normal esophageal epithelium, establishing a precancerous field that predisposes to malignant transformation [20]. Moreover, Liu *et al*. identified early mutations and copy number alterations across non-tumorous, intraepithelial neoplastic, and tumor tissues, suggesting potential genomic biomarkers for early ESCC detection [5].

Recent studies in other cancers have underscored the clinical relevance of NAT-based molecular profiling. In breast cancer, two NAT-derived transcriptomic subtypes were found to correlate with tumor aggressiveness and patient survival [21]. In hepatocellular carcinoma (HCC), three NAT-related molecular subtypes were identified, showing significantly different survival outcomes [22]. At the proteomic level, Zhu *et al*. identified two NAT-based HCC subtypes that were linked to overall survival and recurrence [23]. Ni *et al*. further demonstrated that gastric cancer subtypes defined by NAT proteomes—rather than by tumor proteomes—were more predictive of clinical outcomes [24]. Similarly, Liao *et al*. reported three liver cancer NAT proteomic subtypes associated with recurrence, highlighting metabolic dysregulation as a key driver of NAT heterogeneity and proposing metabolic inhibition as a potential strategy to prevent postoperative relapse [25]. Despite these important advances, proteomic studies simultaneously examining true Healthy, NAT, and Tumor tissues in ESCC remain scarce. Most previous work has treated NAT as a “normal” reference, potentially overlooking early proteomic alterations that drive tumor initiation. Incorporating Healthy tissues into analyses across the Healthy–NAT–Tumor continuum provides a powerful framework to capture early dysregulated proteins and to identify biomarkers for early diagnosis and risk prediction.

In this study, we integrated proteomic data from 20 Healthy esophageal tissues with our previously reported data of 124 paired NAT–Tumor samples [7]. This comprehensive design allowed us to map proteomic alterations across the entire Healthy–NAT–Tumor continuum. By identifying early proteomic changes in NATs relative to Healthy tissues and tracking progressive protein dysregulation along this continuum, we uncovered molecular insights into ESCC evolution. Furthermore, we defined clinically relevant proteomic and immune subtypes, identified potential protein biomarkers, and developed an enhanced prognostic model. Collectively, these findings extend beyond conventional Tumor–NAT comparisons and provide valuable insights for early detection, patient stratification, and therapeutic development.

## Results

### Proteomic analysis of Healthy esophageal tissue samples

**Figure 1:**
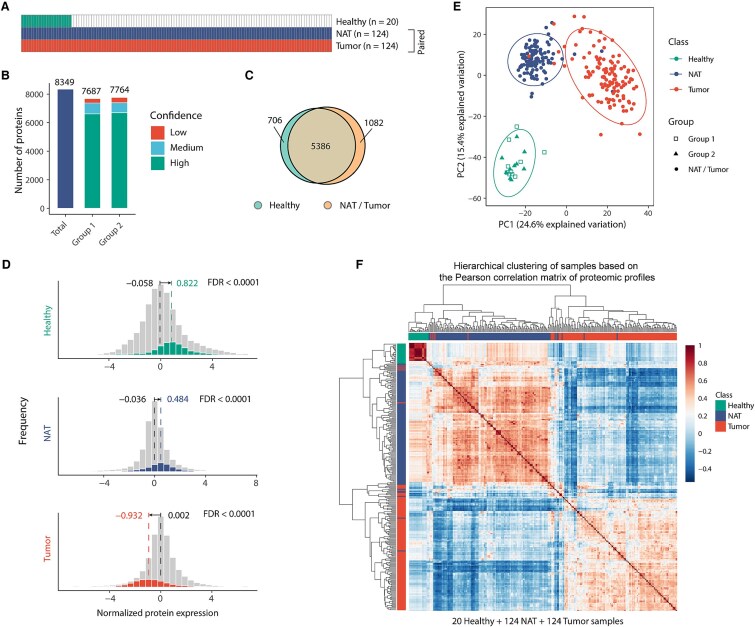
Proteomic landscape of Healthy, NAT, and Tumor tissues in ESCC. (**A**) Overview of sample numbers across Healthy, NAT, and Tumor groups included in the proteomic analysis. (**B**) Distribution of quantified proteins at different confidence levels in Healthy tissues. (**C**) Venn diagram showing overlap of high-confidence proteins quantified in all 20 Healthy samples (Healthy5) and in all NAT/Tumor samples (Prot5). (**D**) Distribution of expression values for all proteins and esophagus-specific proteins across sample classes. Median expression values are indicated for each group. FDRs were calculated using 1D annotation enrichment. (**E**) PCA of TMT proteomic data distinguishing the three sample classes. Two TMT batches for the 20 Healthy samples are indicated by different shapes. Ellipses denote 0.9 confidence intervals for each class. (**F**) Hierarchical clustering of 20 Healthy, 124 NAT, and 124 Tumor samples based on their global proteomic similarity. Pairwise Pearson correlation coefficients were first computed between samples using their protein expression profiles, generating a sample-by-sample correlation matrix (268 × 268). This correlation matrix was then subjected to hierarchical clustering using ‘Euclidean distance’ and ‘complete linkage.’ Samples with similar overall proteomic patterns cluster together, reflecting global similarities across Healthy, NAT, and Tumor tissues.

We performed isobaric tandem mass tag (TMT)-based proteomic profiling of 20 Healthy esophageal tissue samples, using the same protein quantification workflow as in our previous study of 124 paired NAT and Tumor samples [7]. A total of 8349 proteins were identified, and 7149 were quantified [false discovery rate (FDR) < 1% at both the peptide and protein levels] (Fig. 1A, B, and Supplementary Fig. S1A and Supplementary Table S1A) [26]. Among these, 7077 proteins were quantifiable in at least half of the samples, and 6092 were consistently quantified across all samples (Supplementary Fig. S1A and Supplementary Table S1B). Based on these proteins, the pooled internal reference (131C) exhibited highly consistent intensity distributions across TMT batches, with no systematic shift in median reference levels (Supplementary Fig. S1B). Protein expression values were log_2_-transformed and mean-centered to minimize batch effects, following the same normalization approach as in our previous analysis of NAT and Tumor samples. After normalization, expression distributions were homogeneous across the 20 Healthy samples (Supplementary Fig. S1C). Principal component analysis (PCA) and hierarchical clustering using housekeeping genes confirmed that Healthy samples did not segregate by batch but largely overlapped with NAT samples, while Tumor samples formed a distinct cluster (Supplementary Fig. S1D and E).

Across all 20 Healthy, 124 NAT, and 124 Tumor samples, a total of 5386 proteins were consistently quantified (Fig. 1C). Analysis of esophagus-specific proteins from the Human Protein Atlas [27] showed that 268 proteins had significantly higher median expression in Healthy and NAT samples compared to the overall protein background, but were reduced in Tumor samples (annotation enrichment [28], FDR = 0.02) (Fig. 1D). These findings indicate that the TMT-based approach successfully captured the esophagus-specific proteome, especially in Healthy samples (Supplementary Fig. S2). Although NAT samples also showed enrichment of the esophagus-specific proteome, expression levels were slightly lower than those in Healthy samples, whereas Tumor samples exhibited a clear loss of esophageal identity.

To assess biological consistency, key proteomic results were independently validated by immunohistochemistry (Supplementary Fig. S3). Two previously reported ESCC-downregulated proteins, CRNN [29, 30] and DSC2 [31], displayed a gradual decrease in expression from Healthy to NAT to Tumor samples in the TMT-based proteomic data (Supplementary Fig. S3D and H). Consistent with these findings, immunohistochemical staining confirmed that CRNN expression was highest in normal epithelium and decreased along the pathological progression from normal epithelium to low- and high-grade intraepithelial neoplasia and ESCC (Supplementary Fig. S3A–C). DSC2 showed a similar pattern (Supplementary Fig. S3E–G). *Post hoc* computational batch correction introduced artifacts, distorting tissue-specific proteomic patterns (Supplementary Fig. S4) and producing over-correction inconsistent with independent immunohistochemical (IHC) validation (Supplementary Fig. S5). Therefore, computational batch correction was not applied.

Based on the 5386 consistently quantified proteins, PCA and hierarchical clustering clearly separated Healthy samples from NAT and Tumor samples, indicating a distinct global proteomic profile in Healthy esophageal tissues (Fig. 1E and Supplementary Fig. S1F). This result aligns with transcriptomic analyses by Aran *et al*., which demonstrated that normal tissues cluster separately from adjacent normal and tumor tissues across multiple cancer types, including BRCA, COAD, and LIHC, among others [14]. Correlation-based sample-level similarity analysis of the global proteomic profiles further confirmed that Healthy, NAT, and Tumor samples each possess unique expression signatures. NAT samples were more similar to Healthy samples than to Tumor samples, supporting the concept that NAT represents an intermediate molecular state (Fig. 1F) [14].

### Functional analysis of NAT and Tumor samples compared to Healthy samples

**Figure 2:**
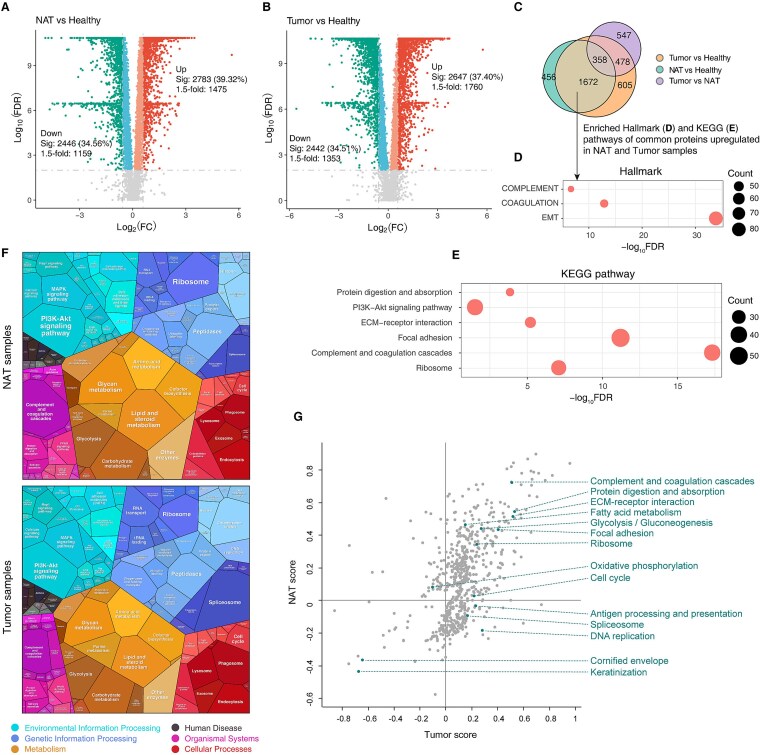
Proteomic alterations in NAT and Tumor tissues relative to Healthy controls. (**A**) Volcano plot showing proteins up- or downregulated in NATs relative to Healthy tissues. Proteins with BH-adjusted *P* < .01 are considered significant, and those with BH-adjusted *P* < .01 and fold change >1.5 are highlighted. Nonsignificant proteins are also shown. *P* values were calculated using a two-sided Wilcoxon rank-sum test. (**B**) Volcano plot showing proteins up- or downregulated in Tumors relative to Healthy tissues. Significance and fold-change thresholds are as described in (**A**). (**C**) Venn diagram displaying the numbers of dysregulated proteins identified from comparisons among Healthy, NAT, and Tumor tissues. (**DE**) Enriched hallmark gene sets (**D**) and KEGG pathways (**E**) of proteins commonly upregulated in NATs and Tumors compared with Healthy tissues. (**F**) Functional categories elevated in NATs (top) and Tumors (bottom) relative to Healthy tissues, visualized using Proteomaps. Each polygon represents a single KEGG pathway, with area proportional to the fold change of its constituent proteins. (**G**) Two-dimensional annotation enrichment analysis of NAT and Tumor proteomes (FDR q-value <0.02).

We next identified differentially expressed proteins in NAT and Tumor samples relative to Healthy controls. Among the 7077 quantifiable proteins (Healthy4; Supplementary Table S1B), 2783 (39.3%) were upregulated and 2446 (34.6%) downregulated in NAT samples (BH-adjusted *P* < .01, Wilcoxon rank-sum test). Of these, 1475 upregulated and 1159 downregulated proteins showed more than 1.5-fold changes (Fig. 2A and Supplementary Table S2A). In Tumor samples, 2647 (37.4%) proteins were upregulated and 2442 (34.5%) were downregulated, with 1760 and 1353 exceeding the 1.5-fold threshold, respectively (Fig. 2B and Supplementary Table S2B). Comparison with previous analyses of Tumor versus NAT differences [6] revealed 2733 newly dysregulated proteins, including 1672 shared by both NAT and Tumor tissues (Fig. 2C). Functional enrichment analysis of co-upregulated proteins (*n* = 1182) highlighted key pathways such as epithelial–mesenchymal transition, complement and coagulation cascades, focal adhesion, and ribosome biogenesis (Fig. 2D and E). These results indicate that although NAT tissues appear histologically normal, their proteomes already exhibit Tumor-like molecular alterations when compared with Healthy tissues.

To examine global proteomic changes in NATs and Tumors, we generated proteomaps based on Kyoto Encyclopedia of Genes and Genomes (KEGG) pathway annotations [32]. The overall proteomic landscapes of NATs and Tumors were strikingly similar, with NATs enriched in metabolic pathways and Tumors showing stronger activation of genetic information processing (Fig. 2F and Supplementary Fig. S6). Immune-related pathways, such as complement and coagulation cascades, were more prominent in NATs, whereas cell cycle-related pathways dominated in Tumors. A 2D annotation enrichment analysis [28] confirmed a strong correlation between NATs and Tumors (R = 0.51; *P* = 9.7 × 10^−16^). Both groups were enriched in pathways including complement and coagulation cascades, protein digestion, ECM–receptor interaction, fatty acid metabolism, and glycolysis, while being depleted in keratinization and cornified envelope pathways (FDR < 0.02) (Fig. 2G and Supplementary Table S2C). In contrast, DNA replication and spliceosome pathways were enriched in Tumors but depleted in NATs, underscoring their functional divergence.

### Proteomic subtype of ESCC defined by the NAT proteome

**Figure 3:**
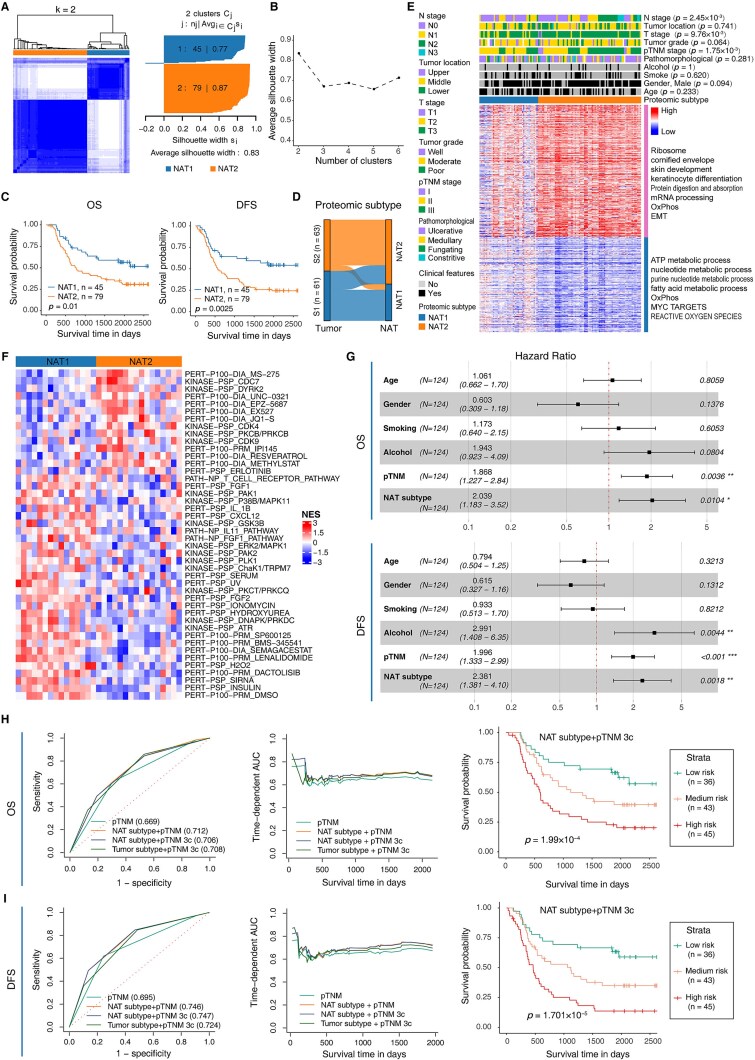
Proteomic subtypes of NAT tissues defined by integrative proteomic analysis. (**A**) Consensus clustering of 124 NAT samples identified two robust clusters (*k* = 2). Left: consensus matrices; right: silhouette width plot. (**B**) Average silhouette width for each *k* value. (**C**) Kaplan–Meier curves for overall survival (OS, left) and disease-free survival (DFS, right) of NAT1 and NAT2 subtypes. *P* values were calculated using a two-sided log-rank test. (**D**) Sankey diagram showing the correspondence between proteomic subtypes defined by NATs and those defined by Tumors. (**E**) Heatmap showing relative protein abundance of differentially expressed proteins between NAT2 and NAT1 (BH-adjusted *P* < .01; fold change >1.5 or < 0.67). The top panel shows associations between NAT subtypes and clinicopathological features. Representative GO biological processes enriched in these proteins are indicated on the right. *P* values were determined using the chi-squared test. (**F**) Heatmap of normalized enrichment scores (NES) for phosphosite-specific signatures significantly dysregulated between NAT1 and NAT2 (unpaired two-sided Student’s t-test, *P* < .05). (**G**) Multivariable Cox regression analysis of NAT subtypes showing hazard ratios (HR, 95% CI) and *P* values for OS (top) and DFS (bottom). Significance levels: *P* < .05 (^*^), < .01 (^**^), < .001 (^***^). (**HI**) Prognostic performance of NAT subtypes for OS (**H**) and DFS (**I**). Left: area under the time-dependent ROC curve (TDROC) at 5 years. Middle: TDROC curves over time. Right: Kaplan–Meier survival curves for three patient groups stratified by the combined “NAT subtype + pTNM 3c” model (*P* values by two-sided log-rank test).

We next investigated whether the NAT proteome could define clinically relevant ESCC subtypes, similar to the Tumor proteome [7]. Consensus clustering of the top 25% most variable proteins identified two major subtypes, NAT1 (*n* = 45) and NAT2 (*n* = 79) (Fig. 3A, B, and  Supplementary Fig. S7A and Supplementary Table S3A). Patients with the NAT2 subtype showed significantly poorer overall survival (OS, *P* = .01) and disease-free survival (DFS, *P* = .0025) compared with NAT1 (Fig. 3C). NAT subtypes were largely concordant with the Tumor subtypes defined by Liu *et al*. [7], showing 75.8% consistency (Fig. 3D). This finding supports the notion that tumor-intrinsic molecular features are reflected in adjacent NAT tissues [33]. However, 23 high-risk NAT2 samples corresponded to the low-risk Tumor S1 subtype, suggesting that NAT tissues harbor additional proteomic alterations not captured in Tumor tissues. Importantly, the NAT subtype showed stronger associations with clinical parameters than the Tumor subtype, including N stage (*P* = 2.45 × 10^−3^ versus 0.0496), T stage (*P* = 9.76 × 10^−3^ versus 0.55), and pTNM stage (*P* = 1.75 × 10^−3^ versus 0.20) (Fig. 3E and Supplementary Table S3B) [7].

Differential expression analysis of 6468 quantified proteins revealed 887 upregulated and 1306 downregulated proteins in NAT2 relative to NAT1 (BH-adjusted *P* < .01) (Supplementary Fig. S7B and Supplementary Table S3C). Heatmap visualization showed that NAT2 exhibited more extreme expression profiles, with markedly upregulated and downregulated protein groups. Upregulated proteins were enriched in ribosome and mRNA processing pathways—patterns shared with the Tumor S2 subtype—while novel pathways like keratinization and cornified envelope were also upregulated in NAT2 (Fig. 3E and Supplementary Table S3D). Many proteins in these pathways, including COL5A1 [34], SPRR1A/B [35], and DSC2 [36], have been linked to tumor progression and poor survival in multiple cancers. These findings suggest that similar malignant mechanisms operate in NAT tissues, contributing to the aggressive NAT2 subtype. In addition, NAT2 was also characterized by downregulation of metabolic pathways (e.g. adenosine triphosphate (ATP), nucleotide, fatty acid metabolism) (Fig. 3E and Supplementary Table S3E).

We further examined differences in protein phosphorylation levels between NAT1 and NAT2 subtypes. Among the 31 ESCC patients with quantified phosphorylation data from our previous study [7], 15 belonged to NAT1 and 16 to NAT2 (Supplementary Table S3A). Differential analysis revealed 1330 upregulated and 1664 downregulated phosphorylation sites in NAT2 (*P* < .01, Wilcoxon rank-sum test) (Supplementary Fig. S7C and Supplementary Table S3F). PTM-SEA analysis identified 14 upregulated and 30 downregulated phosphorylation signatures in NAT2 (Fig. 3F). Five kinase–substrate signatures (DYRK2, CDC7, GSK3B, DNAPK/PRKDC, and ATR) and five perturbation signatures (PRM_IPI145, DIA_UNC-0321, DIA_MS-275, IONOMYCIN, and DIA_SEMAGACESTAT) were significantly altered in both NAT2 and the Tumor S2 subtype [7]. These overlapping phosphorylation signatures may represent potential therapeutic targets for patients with NAT2 or Tumor S2 subtypes. In contrast, signaling pathways related to T-cell receptor, IL11, and FGF1 were significantly downregulated in NAT2, indicating suppression of immune-related processes (Fig. 3F).

Given the clinical relevance of the NAT subtype, we further evaluated its prognostic value in ESCC patients. Cox regression analysis demonstrated that NAT subtypes were significantly associated with both OS (hazard ratio = 1.941, *P* = .011) and DFS (hazard ratio = 2.154, *P* = 3.13 × 10^−3^). The NAT subtype remained an independent prognostic factor for OS (*P* = .010) and DFS (*P* = 1.81 × 10^−3^) (Fig. 3G and Supplementary Table S3G). We developed two prognostic models—“NAT subtype + pTNM” and “NAT subtype + pTNM 3c.” Both models improved the 5-year AUCs for OS and DFS prediction compared with the pTNM stage alone (Fig. 3H and I, left). Time-dependent ROC analyses confirmed stable performance across time points (Fig. 3H and I, middle). Notably, the “NAT subtype + pTNM 3c” model achieved significantly better survival stratification than pTNM alone (log-rank *P*: 1.99 × 10^−4^ versus 4.70 × 10^−3^ for OS; 1.70 × 10^−5^ versus 8.80 × 10^−4^ for DFS) (Fig. 3H and I, right). These results highlight the clinical utility of integrating NAT subtypes to refine the prognostic accuracy of the pTNM system.

### Characterization of NAT cellular composition

**Figure 4:**
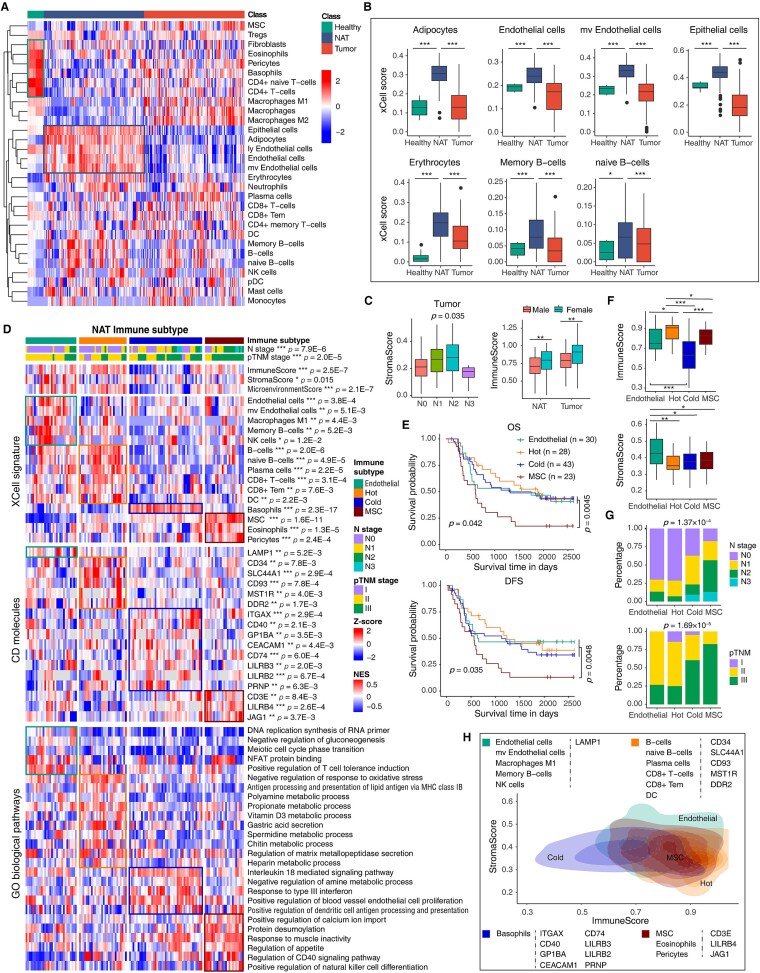
Cellular composition and immune-based subtypes of NAT tissues. (**A**) Heatmap showing xCell-derived enrichment scores for immune and stromal cell types estimated from global proteomic data. Cell types were clustered using hierarchical clustering. (**B**) Boxplots of xCell scores for representative cell types enriched in NATs compared with other tissues. (**C**) Correlations between xCell-derived scores and clinical characteristics. Left: association of StromaScore with N stage. Right: association of ImmuneScore with sex. (**D**) Heatmap depicting cell-type compositions and activities of selected CD molecules and immune pathways across the four immune clusters. *P* values in the top two sections were computed using the Kruskal–Wallis rank-sum test, and those in the remaining sections using two-sided Wilcoxon rank-sum tests (one cluster versus others). (**E**) Kaplan–Meier survival curves for overall survival (OS, top) and disease-free survival (DFS, bottom) across immune clusters (*P* values by two-sided log-rank test). (**F**) Boxplots showing ImmuneScore (top) and StromaScore (bottom) distributions among the four immune clusters. (**G**) Histograms displaying the proportions of patients with different N (top) and pTNM (bottom) stages across immune clusters (*P* values by χ^2^ test). (**H**) Contour plots illustrating the 2D density distributions based on ImmuneScore (x-axis) and StromaScore (y-axis) for each immune cluster. For each cluster, enriched cell types and upregulated CD molecules are annotated. *P* values in (**B**), (**C**, right), and (**F**) were calculated using two-sided Wilcoxon rank-sum tests; *P* values in (**C**, left) were calculated using the Kruskal–Wallis rank-sum test. Significance levels: *P* < .05 (^*^), < .01 (^**^), < .001 (^***^).

Previous pan-cancer transcriptomic studies have shown that NAT tissues differ from both Healthy and Tumor tissues in cellular composition [14]. To examine this in ESCC, we used xCell [37] to estimate the enrichment of 30 immune and stromal cell types across Healthy, NAT, and Tumor samples (Supplementary Table S4). Hierarchical clustering revealed clear compositional differences among the three tissue types (Fig. 4A). Healthy tissues were enriched in fibroblasts, eosinophils, pericytes, basophils, CD4^+^ naïve T cells, and CD4^+^ T cells. In contrast, NATs showed higher levels of adipocytes, endothelial cells, epithelial cells, erythrocytes, memory B cells, and naïve B cells (Fig. 4B and Supplementary Fig. S8A), whereas macrophages were depleted (Supplementary Fig. S8B). Fibroblast abundance decreased progressively from Healthy to NAT to Tumor, while mesenchymal stem cells (MSCs) showed the opposite trend (Supplementary Fig. S8C). NATs displayed the lowest immune scores but the highest stroma scores (Supplementary Fig. S8D). Stroma scores correlated with N stage in Tumors, and immune scores were higher in females than in males in both NAT and Tumor tissues (Fig. 4C), consistent with prior studies [38].

Consensus clustering of xCell-derived profiles identified four clinically relevant immune subtypes in NATs: Endothelial (*n* = 30), Hot (*n* = 28), Cold (*n* = 43), and MSC (*n* = 23) (Fig. 4D). Both OS (log-rank *P* = .042) and DFS (*P* = .035) differed among these subtypes, with the MSC subtype showing the worst outcomes (OS *P* = .0045, DFS *P* = .0048; Fig. 4E). Hot and Cold subtypes had the highest and lowest immune scores, respectively, whereas the Endothelial subtype exhibited the highest stroma scores (Fig. 4D and F). All subtypes were significantly associated with N stage (χ^2^-test, *P* = 1.37 × 10^−4^) and pTNM stage (χ^2^-test, *P* = 1.69 × 10^−5^) (Fig. 4D and G).

Cell-type enrichment analyses revealed distinct characteristics for each subtype. Endothelial NATs were enriched for endothelial cells, M1 macrophages, memory B cells, and NK cells. Hot NATs contained abundant B cells, plasma cells, CD8^+^ T cells, and dendritic cells. High CD93 expression in this subtype suggested potential sensitivity to anti-CD93 therapy [39–41] (Fig. 4D and H). Hot NATs also showed upregulation of metabolic pathways, including polyamine and spermidine metabolism (Fig. 4D). Cold NATs exhibited enrichment of basophils and upregulation of CD molecules such as ITGAX and CD40 (Fig. 4D). The presence of basophils in the Cold subtype implies a role for basophils in NAT immune regulation, consistent with previous observations in early lung adenocarcinoma and non-involved lung tissue [42].

The MSC subtype included more advanced patients (Fig. 4G). It was characterized by enrichment of MSCs, eosinophils, and pericytes, along with upregulation of CD3E, LILRB4, and JAG1 (Fig. 4D and H). Based on xCell enrichment scores, all three cell types stratified ESCC patients into two groups with significant survival differences—for OS (log-rank *P* = 3.0 × 10^−5^, .011, and 1.6 × 10^−4^, respectively; Supplementary Fig. S8E) and for DFS (log-rank *P* = 8.4 × 10^−5^, 3.6 × 10^−3^, and 3.6 × 10^−4^, respectively; Supplementary Fig. S8F). MSCs, which promote tumor progression in many cancers [43–48], increased progressively from Healthy to NAT to Tumor tissues (Supplementary Fig. S8C). Higher MSC activity in Tumors was associated with worse OS (log-rank *P* = .013; Supplementary Fig. S8G) and DFS (log-rank *P* = 7.4 × 10^−3^; Supplementary Fig. S8H). These findings highlight MSCs as key contributors to ESCC progression and potential therapeutic targets.

Given the identification of two proteomic NAT subtypes, we next asked whether these subtypes differ in cellular composition and whether they more closely resemble Healthy or Tumor tissues. Hierarchical clustering of xCell enrichment scores across Healthy, NAT, and Tumor samples did not reveal a clear pattern in which NAT1 clustered with Healthy tissues or NAT2 with Tumors (Supplementary Fig. S9A). This suggests that the two NAT proteomic subtypes cannot be simply interpreted as “Healthy-like” or “Tumor-like” at the level of overall cellular composition. We therefore performed a differential analysis of xCell enrichment scores between NAT2 and NAT1. Among the 30 immune and stromal cell types evaluated, only adipocytes and CD4^+^ T cells showed statistically significant differences after multiple testing correction (Supplementary Fig. S9B). Further stratified analyses showed that adipocyte enrichment increased from Healthy samples to NAT1, reached a peak at NAT1, and then declined stepwise from NAT2 to S1 and S2 (Supplementary Fig. S9C). In contrast, CD4^+^ T-cell enrichment displayed the opposite trend, with the lowest levels in NAT1 and a gradual increase across NAT2, S1, and S2 (Supplementary Fig. S9D). These results suggest that, although NAT proteomic subtypes do not globally mirror Healthy or Tumor cellular states, they differ along specific cellular axes that show a continuous trend across NAT and Tumor subtypes.

### Proteomic changes from Healthy to NAT to Tumor

**Figure 5:**
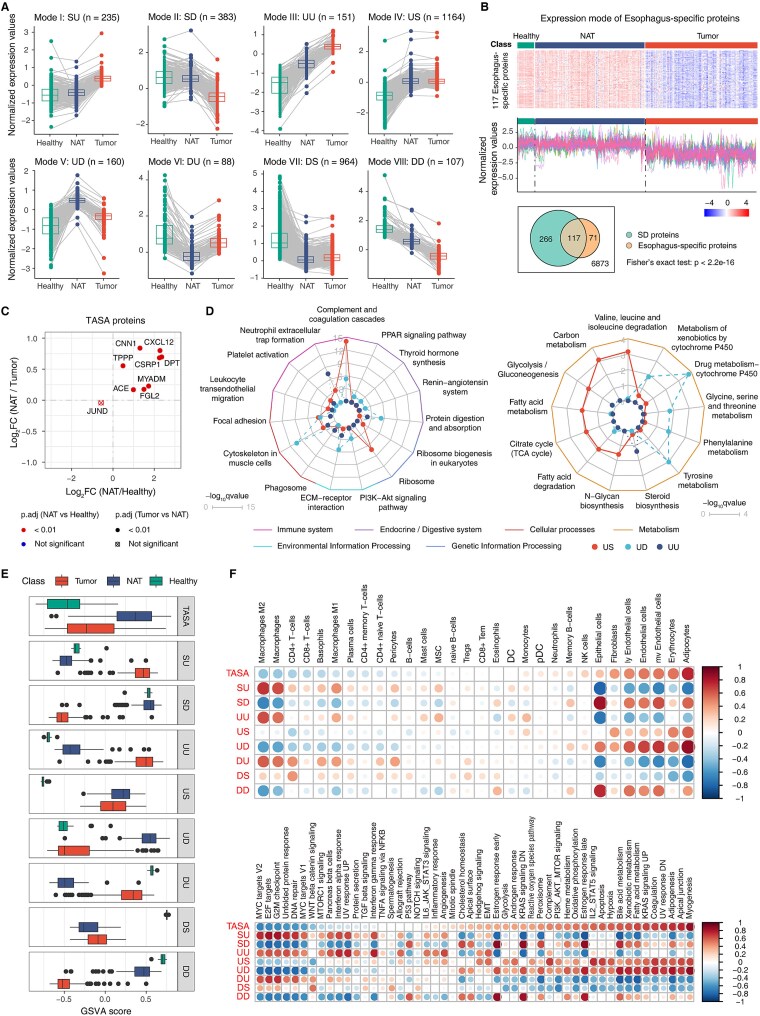
Proteomic and functional alterations across Healthy, NAT, and Tumor tissues. (**A**) Expression patterns of proteins grouped into eight distinct expression modes. Boxplots and line plots depict representative profiles for each mode; numbers in parentheses indicate the number of proteins per mode. The SS mode is not shown. (**B**) Heatmap (top) and line plots (middle) illustrating expression patterns of esophagus-specific proteins across the three tissue types. Bottom: Venn diagram showing the overlap between SD proteins and esophagus-specific proteins. (**C**) Scatter plot of TASA proteins. *P* values were calculated by two-sided Wilcoxon rank-sum tests. The color and shape of each point indicate the significance of the NAT versus Healthy and NAT versus Tumor comparisons, respectively. (**D**) Enrichment analysis of selected KEGG pathways for proteins following US, UD, and UU expression modes. Radial plots represent –log₁₀(*P*) values of enriched pathways. Left: pathways related to biological system processing, cellular and environmental information, and genetic information. Right: metabolic pathways. The complete list of enriched pathways is provided in Supplementary Table S5. (**E**) Boxplots showing GSVA enrichment scores for the TASA signature and for the eight protein expression modes. (**F**) Spearman correlation coefficients between GSVA scores of protein sets and xCell-derived cell-type scores. (**G**) Spearman correlation coefficients between GSVA scores of protein sets and hallmark gene sets.

To trace proteomic changes across the Healthy–NAT–Tumor continuum, we classified proteins based on differential expression patterns. Each protein was categorized as upregulated (U), downregulated (D), or stable (S), resulting in nine groups: SU (*n* = 235), SD (*n* = 383), UU (*n* = 151), US (*n* = 1164), UD (*n* = 160), DU (*n* = 88), DS (*n* = 964), DD (*n* = 107), and SS (*n* = 3621) (Fig. 5A).

Proteins with SU or SD patterns showed a “Healthy-like” tendency in NATs, remaining stable from Healthy to NAT but changing significantly in Tumors. Esophagus-specific proteins were enriched in SD proteins (Fisher’s exact test, *P* < 2.2 × 10^−16^). Among these, 117 proteins were preserved in NATs but markedly lost in Tumors, including KRT4, KRT6A, KRT6B, GBP6, TGM1, and TGM3—key biomarkers of normal esophageal epithelium (Fig. 5B and Supplementary Fig. S10A). This pattern likely explains the morphological similarity between NAT and Healthy tissues and their divergence from Tumors. SD proteins were associated with epidermis development and keratinocyte differentiation, confirming their downregulation in Tumors (Supplementary Figs S11 and S12B and Supplementary Table S5B). In contrast, SU proteins were enriched in pathways like DNA replication and cell cycle, suggesting their activation during tumorigenesis (Supplementary Figs S11 and S12B and Supplementary Table S5A). Hallmark enrichment analysis also revealed enrichment of E2F targets, G2M checkpoint, and MYC target gene sets among SU proteins, consistent with the findings of Aran *et al*. that these pathways exhibit a “Healthy-like” pattern in NATs (Supplementary Table S5A) [14].

Proteins with US, UD, or UU patterns were upregulated early, already at the Healthy-to-NAT transition. UD proteins correspond to tumor-adjacent specific activation (TASA) genes previously described by Aran *et al*. [14]. Of the 18 TASA genes identified in that study, protein products of 9 were quantified here, and 8 (except JUND) displayed the same UD pattern—upregulated in NATs relative to Healthy tissues but downregulated in Tumors relative to NATs (Fig. 5C). This strong concordance between proteomic and transcriptomic data underscores the reliability of TASA genes as early biomarkers. UD proteins were enriched in immune-related pathways, including complement and coagulation cascades, suggesting their early involvement in tumor initiation (Fig. 5D, Supplementary Fig. S12C, and  D and Supplementary Table S5C–E). Complement components such as C1QC [49], C2 [50], C3 [51], C4A [52], C4BPA [53, 54], and CFB [55–57] were dysregulated early in NATs, linking the complement system to cancer progression (Supplementary Fig. S13). Metabolic reprogramming was also evident in NATs, with glycolysis and fatty acid metabolism pathways upregulated, reflecting a “Tumor-like” metabolic state (Fig. 5D and Supplementary Table S5D). UU proteins exhibited a stepwise activation pattern from Healthy to NAT to Tumor. We validated this pattern using 22 reported ESCC biomarkers, finding that 9 oncogenic proteins followed the same gradient (Supplementary Table S6 and Supplementary Fig. S10B). However, some markers such as CALR and NCL displayed a DU pattern, showing decreased expression in Tumors (Supplementary Fig. S10C). Two tumor suppressors, ANXA1 and PRDX2, followed the SD pattern (Supplementary Fig. S10D). Although both were downregulated in Tumors—consistent with prior reports [58]—their diagnostic utility for early ESCC should be interpreted with caution, as no decline occurred from Healthy to NAT. Proteins with the DD pattern were associated with keratinization and epidermal development (Supplementary Fig. S12F), suggesting potential biomarker value (Supplementary Fig. S10E). Enrichment analysis further indicated that UU proteins were involved in epithelial–mesenchymal transition (EMT) and extracellular matrix organization, pathways that were also enriched among SU and US proteins, highlighting their involvement in the transition from Healthy to NAT to Tumor (Supplementary Table S5A, C, and D).

To link proteomic alterations with cellular context, we performed correlation analysis using gene set variation analysis (GSVA) enrichment scores [59] (Fig. 5E and F). Consistent with previous transcriptomic findings [14], both TASA and UD scores were strongly associated with adipocytes and endothelial cells (Fig. 5F). Adipocytes serve as alternative energy sources for tumor cells [60, 61], and their enrichment aligns with early metabolic changes in NATs. Notably, the progressive changes in adipocyte and CD4^+^ T-cell enrichment observed across NAT and Tumor subtypes (Supplementary Fig. S9C and D) are consistent with their strong associations with early proteomic alterations in NATs, further linking cellular context to disease-related proteomic remodeling. Correlation with hallmark gene sets confirmed that TASA and UD scores were strongly associated with adipogenesis and fatty acid metabolism—pathways that promote angiogenesis and pro-tumor immune phenotypes (Fig. 5F) [62]. Proteins in SU, DU, and UU groups were primarily correlated with M2 macrophages, an alternative activation state associated with tumor initiation, angiogenesis, progression, and metastasis [63]. These protein groups were also significantly associated with hallmarks such as MYC targets, E2F targets, and the G2M checkpoint (Fig. 5F). MYC, a multifunctional transcription factor, regulates nearly 45% of genes driving M2-like macrophage activation and is increasingly recognized as an M2 macrophage marker [64, 65]. It represents a potential therapeutic target in M2-driven tumors. Consistently, SU, DU, and UU proteins were also enriched for TGF-β signaling—a well-established inducer of M2 polarization [66]. Together, these results suggest that early proteomic alterations in NATs are closely linked to metabolic reprogramming, complement activation, and immune modulation. The interplay among these pathways and specific cell types, particularly adipocytes, endothelial cells, and M2 macrophages, likely plays a critical role in ESCC pathogenesis and warrants further investigation.

### US proteins accurately predict survival risk in ESCC

We investigated the survival associations of proteins with distinct expression patterns. Based on eight expression-pattern-defined protein sets (SU, SD, US, UD, UU, DU, DS, and DD) and their union (“All”), we systematically evaluated their prognostic potential, with particular attention to US proteins, which are often overlooked in traditional Tumor–NAT analyses but may serve as potent biomarkers due to their upregulation.

**Figure 6:**
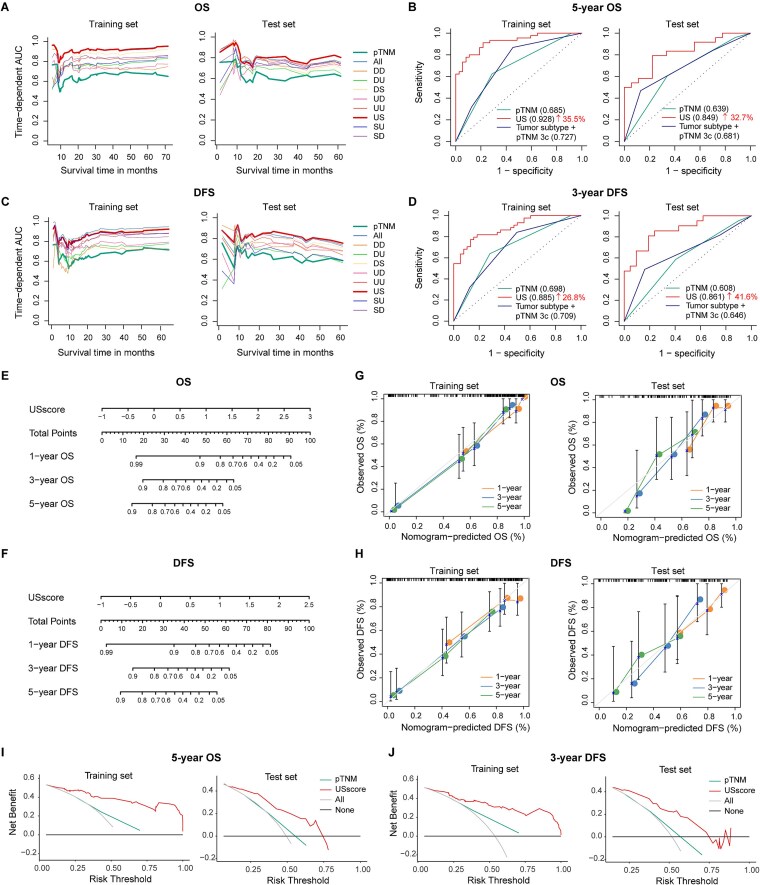
Prognostic performance of protein expression modes in ESCC. (**AC**) Time-dependent AUC curves for OS (**A**) and DFS (**C**). “All”: model integrating proteins from all eight expression modes. (**B**) Five-year ROC curves for OS. (**D**) Three-year ROC curves for DFS. Numbers in parentheses indicate AUC values. “Tumor subtype + pTNM 3c”: reference model from Liu *et al*. [7]. (**EF**) Nomograms for OS (**E**) and DFS (**F**) prediction based on Ridge-Cox models using US proteins (USscore, model-derived risk score). (**GH**) Calibration plots for the nomograms in (**E**) and (**F**). (**IJ**) Decision curve analyses for 5-year OS (**I**) and 3-year DFS (**J**) in the training (left) and test (right) cohorts.

A total of 124 ESCC patients were randomly divided into a training cohort (*n* = 82) and a test cohort (*n* = 42), with no significant differences in clinical characteristics (*P* > .05; Supplementary Table S7A). Using these nine protein sets, we built Ridge-Cox models [67] and compared their predictive performance with the pTNM staging system. As summarized in Supplementary Table S7B, all pattern-specific models achieved comparable or improved C-indices relative to pTNM for both OS and DFS. Among them, the “US” model achieved the highest C-index in the independent test set for OS (0.731), whereas the “All” model performed best in the training set but slightly worse in the test set, indicating potential overfitting. Time-dependent AUC (tdAUC) analysis further confirmed that the US model consistently outperformed other models over time, achieving 5-year AUCs of 0.928 in the training set and 0.849 in the test set, representing 35.5% and 32.7% improvements over pTNM (Fig. 6B and Supplementary Table S7C).

To determine whether a smaller protein subset could achieve similar predictive accuracy, we applied three feature-selection–based survival algorithms—Lasso-Cox [67], sslasso [6, 8], and glmboost [68]. The US model achieved the best overall performance, ranking first for Lasso-Cox and sslasso and second for glmboost in the test set (Supplementary Table S7E–G and Supplementary Fig. S14). Although these models outperformed pTNM in the training set, their advantages diminished in the test set compared with Ridge-Cox, indicating that integrating all proteins within each expression mode yields the most robust predictions.

For DFS, the US model again demonstrated the best performance in the test cohort (C-index = 0.735), with 3-year AUCs of 0.885 and 0.861 in the training and test sets, respectively. These results represent 26.8% and 41.6% improvements over pTNM (Fig. 6C and D and Supplementary Table S7B and D).

Nomograms for OS and DFS were developed using Ridge-Cox US model scores (USscore). Calibration curves showed close agreement between predicted and observed outcomes (Fig. 6E–H). Decision curve analysis further demonstrated that adding USscore provided greater clinical benefit than the “All treatment,” “No treatment,” or pTNM models, supporting its clinical applicability (Fig. 6I and J).

Finally, by integrating proteomic data from both Tumor and NAT tissues, we identified 19 US proteins whose expression correlated with OS and DFS (Cox *P* < .01) (Supplementary Fig. S15A). Among these, NIPSNAP1 and MTHFS were associated with worse outcomes, whereas 17 proteins were protective (Supplementary Fig. S15B). Notably, 16 of the 19 proteins have been previously linked to cancer prognosis (Supplementary Table S8), validating their biological and clinical relevance. Together, these findings reinforce the prognostic relevance of US proteins and suggest their potential as early indicators of ESCC initiation and progression.

## Discussion

This study presents a comprehensive proteomic characterization of Healthy, NAT, and Tumor tissues in ESCC, revealing that NATs undergo extensive early proteomic reprogramming compared with Healthy tissues and display substantial alterations relative to Tumors. We identified clinically relevant NAT-based proteomic and immune subtypes, providing new opportunities for patient stratification and potential guidance for immunotherapy. In addition, analysis of eight protein expression patterns uncovered early molecular events that mark the onset of malignant transformation.

Consistent with previous transcriptomic observations [14], NATs, though morphologically similar to Healthy tissues, exhibit profound proteomic remodeling and occupy an intermediate molecular state between Healthy and Tumor tissues. Esophagus-specific proteins were enriched in NATs (Fig. 1D and Supplementary Fig. S2) and were mainly classified into the SD group (Fig. 5B), supporting the structural resemblance of NATs to Healthy tissues. However, NATs also showed Tumor-like molecular traits, including alterations in immune-related and metabolic pathways (Fig. 5D). Among these, the complement system emerged as one of the earliest and most strongly activated pathways (Supplementary Fig. S13). Beyond its classical role in innate immunity, the complement cascade also modulates adaptive immune responses and inflammatory signaling [45]. This finding aligns with prior transcriptomic evidence showing enhanced immune and inflammatory activity in tumor-adjacent tissues [5], suggesting that immune reprogramming precedes visible tumor formation. Early activation of complement and related immune pathways may contribute to a pro-tumorigenic microenvironment through cross-talk with adaptive immunity, chronic inflammation, and stromal remodeling. Moreover, since complement signaling has been proposed as an immunotherapeutic target in several other malignancies, including colon and liver cancers [45–47], its early activation in NATs may represent both an indicator of early tumorigenic changes and a potential therapeutic vulnerability in ESCC.

In parallel with immune and inflammatory activation, extensive metabolic alterations constitute another hallmark of NAT tissues. Our proteomic analysis revealed pronounced upregulation of multiple metabolic pathways (Fig. 5D), indicating that metabolic reprogramming emerges early, even before overt malignant transformation. Similar metabolic rewiring has been widely reported across cancers, where enhanced glycolysis (the Warburg effect), lipid biosynthesis, and amino acid metabolism sustain anabolic growth and cellular survival under metabolic stress [69, 70]. The metabolic remodeling observed in NATs may therefore reflect an adaptive, “pre-malignant” state that facilitates stromal activation and tumor initiation. Furthermore, upregulation of fatty acid metabolism and TCA cycle pathways suggests broad shifts in bioenergetic and biosynthetic processes, consistent with evidence that metabolic intermediates can modulate signaling, immune regulation, and epigenetic remodeling within the tumor niche [71]. Collectively, these findings highlight NAT-associated metabolic reprogramming as both an early molecular signature and a potential therapeutic vulnerability in ESCC.

Our study also identified two proteomic subtypes in NAT tissues, which showed significant associations with OS and DFS. These associations were even stronger than those of Tumor-derived subtypes (Fig. 3C, E, and  G). The “NAT subtype + pTNM 3c” model outperformed the pTNM system in risk stratification, emphasizing the clinical importance of NAT proteomic alterations (Fig. 3H and I). Additionally, four prognostically relevant immune subtypes were detected in NATs but not in Tumors (Fig. 4D and E). This finding suggests that NATs actively shape the peritumoral ecosystem and may provide more accurate prognostic insights than Tumor-based classifications, a pattern also observed in hepatocellular and gastric cancers [23–25]. We also identified key progression-related proteins—such as COL5A1, SPRR1A/B, and DSC2—and enriched stromal cell types including MSCs within NATs. These results support the field cancerization hypothesis [10, 11], which posits that histologically normal tissues adjacent to tumors undergo molecular and metabolic changes that predispose them to malignancy. Remodeling of the extracellular matrix, immune signaling, and metabolic processes in NATs may therefore represent a premalignant state that facilitates tumor progression [72, 73].

Across Healthy–NAT–Tumor tissues, we observed eight distinct protein expression patterns, including UD proteins that mirrored the transcriptional activation of TASA genes in NATs [14]. Previously proposed ESCC biomarkers were also reevaluated, confirming the reliability of those following UU or DD expression modes, while indicating that several candidates may require reassessment (Supplementary Fig. S10B–E). Notably, US proteins—largely overlooked in prior studies—captured early molecular alterations in NATs, particularly immune activation and metabolic remodeling (Fig. 5D). These proteins demonstrated strong prognostic potential, with the US model outperforming the pTNM system (Fig. 6). By integrating NAT and Tumor proteomes, we further identified 19 robust prognostic proteins associated with both OS and DFS across tissue types (Supplementary Table S8).

In conclusion, this proteomic study provides a multidimensional view of ESCC pathogenesis, revealing early immune and metabolic activation in NATs. By defining clinically meaningful proteomic and immune subtypes, we offer new insights into early tumorigenic processes, patient stratification, and therapeutic development in ESCC.

## Materials and Methods

### Clinical sample and proteomic data generation

A total of 124 paired NAT and Tumor tissue samples were collected from Shantou Central Hospital as described previously [7], and 20 Healthy esophageal tissue samples were obtained from healthy volunteers at the Affiliated Cancer Hospital of Shantou University Medical College in 2019 [26]. All tissue specimens were snap-frozen in liquid nitrogen within 10 minutes after surgical resection and stored at −80°C until further analysis. Written informed consent was obtained from all participants. All procedures involving human participants were approved by the Ethics Committees of the participating institutions and conducted in accordance with the Declaration of Helsinki. Proteomic analysis of the 20 Healthy samples was performed using the same TMT-based quantitative workflow as previously applied to the 124 paired NAT–Tumor samples [7]. Healthy samples were analyzed in 2 TMT 11-plex experiments. In each experiment, a pooled internal reference—prepared by combining equal amounts of peptides from 60 paired NAT–Tumor samples—was labeled with the 131C channel, while 10 individual Healthy samples were assigned to the remaining TMT channels. The same pooled internal reference (131C) was included in all TMT batches to enable cross-batch normalization and comparability. Detailed protocols for tissue processing, liquid chromatography, and tandem mass spectrometry (LC–MS/MS) are provided in Supplementary Methods.

### Immunohistochemistry

IHC staining was performed on esophageal endoscopic submucosal dissection (ESD) specimens obtained from 23 patients with continuous esophageal lesions, including normal esophageal epithelium, low-grade intraepithelial neoplasia (LGIN), high-grade intraepithelial neoplasia (HGIN), and ESCC. This additional IHC study was approved by the Ethics Committee of Shantou University Medical College (SUMC-2024-070) and conducted in accordance with the Declaration of Helsinki.

ESD tissues were fixed in 10% neutral-buffered formalin, dehydrated, embedded in paraffin, and sectioned into 3-μm slices. For IHC staining, sections were deparaffinized in xylene and rehydrated through graded alcohols as described previously [7, 26]. Antigen retrieval was performed using either citrate buffer (pH 6.0) or EDTA buffer (pH 9.0), followed by cooling at room temperature for at least 30 minutes. Endogenous peroxidase activity was blocked by incubation with 3% hydrogen peroxide for 10 minutes. Sections were then incubated overnight at 4°C with primary antibodies against CRNN (1:400, 11799-1-AP, Proteintech, Wuhan, China) or DSC2 (1:2000, 13876-1-AP, Proteintech, Wuhan, China). Immunostaining was carried out using a polymer-based detection system (MD012-N1, Dongguan Yiben Biotechnology Co., Ltd., China), with diaminobenzidine (DAB) as the chromogen, followed by hematoxylin counterstaining. Slides processed without primary antibody were used as negative controls.

IHC staining was independently evaluated under light microscopy by an experienced pathologist. Protein expression levels were semiquantitatively scored based on staining intensity and the percentage of positive cells in each histological region. Staining intensity was graded as 0 (none), 1 (weak), 2 (moderate), or 3 (strong). The percentage of positive cells was scored as 0 (0%–5%), 1 (6%–25%), 2 (26%–50%), 3 (51%–75%), or 4 (>75%). The final IHC score was calculated by multiplying the two subscores, yielding a total score ranging from 0 to 12.

### Global proteomic data analysis

Protein expression ratios were calculated relative to the internal reference, log_2_-transformed, and mean-centered. Five datasets were generated based on quantification frequency and confidence criteria (Supplementary Fig. S1A). For comparative analysis across tissue types, PCA and hierarchical clustering were performed in R (v4.4.0) using proteomic data from 5386 proteins consistently quantified across all samples (20 Healthy, 124 NAT, and 124 Tumor samples). PCA was conducted to assess global variance patterns among tissue types, with confidence intervals estimated using the *ggbiplot* R package (v0.6.2). Hierarchical clustering based on protein expression profiles was performed using ‘Euclidean distance’ and ‘complete linkage,’ enabling evaluation of global proteomic similarity across samples (Supplementary Fig. S1F).

#### Housekeeping gene-based analysis

To further evaluate potential batch-related effects on global expression patterns, we analyzed housekeeping (HK) genes, which are expected to remain relatively stable across tissues and experimental conditions. HK genes were obtained from the Housekeeping and Reference Transcript Atlas (HRT Atlas v1.0; www.housekeeping.unicamp.br) [74]. The intersection of reference genes annotated for mucosa, muscularis, and gastroesophageal junction tissues was used to define the HK gene set. The top 40 HK genes ranked by expression stability were selected for downstream analyses. PCA based on these genes revealed that Healthy and NAT samples clustered together, whereas Tumor samples formed a distinct cluster, indicating the absence of systematic technical separation between Healthy and NAT tissues. Hierarchical clustering using the same HK gene set was performed with the *pheatmap* R package (v1.0.13) under the following parameters: *clustering_distance_rows* = “*euclidean,*” *clustering_distance_cols* = “*euclidean,*” and *clustering_method* = “*complete.*”

#### Correlation-based sample-level similarity analysis

For the sample-level similarity analysis (Fig. 1F), pairwise Pearson correlation coefficients were calculated across samples based on their global proteomic expression profiles, generating a sample-by-sample correlation matrix. Hierarchical clustering was then performed on this matrix using ‘Euclidean distance’ and ‘complete linkage.’ The resulting dendrogram reflects global similarities in proteomic expression patterns across all samples.

#### Batch correction assessment

To evaluate the potential impact of computational batch correction on protein expression patterns, two commonly used methods—*removeBatchEffect* (*limma* package, v3.66.0) and *ComBat* (*sva* package, v3.58.0)—were applied under two different batch definitions: (i) two batches (Healthy versus NAT/Tumor), and (ii) 27 batches, corresponding to individual TMT experiments. Batch-corrected expression levels of representative proteins (CRNN and DSC2) were compared across tissue types under each strategy (Supplementary Fig. S4). Statistical significance was assessed using two-sided Wilcoxon rank-sum tests. These analyses were performed to examine whether *post hoc* correction introduced artificial shifts inconsistent with independent experimental validation.

#### Differential expression analysis

Differential expression analysis was conducted using nonparametric statistical tests, with significance defined as a Benjamini–Hochberg adjusted *P*-value <.01 and an absolute fold change >1.5.

### NAT proteomic subtype analysis

NAT proteomic subtypes were identified by consensus clustering of the top 25% most variable proteins, using the R package *ConsensusClusterPlus* [75]. Differential expression and phosphorylation between subtypes were assessed using the Wilcoxon rank-sum test. For completeness, unsupervised clustering analyses were also applied to the Healthy control proteomes; however, no biologically interpretable subtypes were detected (Supplementary Fig. S16).

### Cellular composition and functional analysis

Tissue cellular composition was deconvoluted from proteomic data using *xCell* [37], a single-sample Gene Set Enrichment Analysis (*ssGSEA*)-based approach [76] that has been successfully applied to proteomic datasets [77–79]. Immune subtypes were identified by consensus clustering, and survival differences were evaluated by the log-rank test. Proteomaps were generated using the *Proteomaps* web tool (www.proteomaps.net) to visualize functional categories enriched among proteins upregulated in NATs or Tumors samples [32]. Two-dimensional annotation enrichment analysis was conducted with *Perseus* (v2.0.11) [28, 80]. Functional enrichment analyses were performed using *Metascape* and *clusterProfiler*, with statistical significance assessed by the hypergeometric test. PTM-SEA was executed with ssGSEA2.0 (https://github.com/broadinstitute/ssGSEA2.0) against the PTMsigDB (v1.9.0; https://proteomics.broadapps.org/ptmsigdb/) to identify enriched posttranslational modification signatures [81].

### Survival analysis and prognostic model construction

OS and DFS differences were assessed using Kaplan–Meier survival curves and Cox regression. We constructed two primary models based on NAT proteomic subtypes: (i) The “NAT subtype+pTNM” model, a Cox PH model integrating the NAT subtype and pTNM stage; (ii) the “NAT subtype+pTNM 3c” staging system, derived by applying *k*-means clustering (*k* = 3) to risk scores from the first model. The three resulting clusters were designated as low-, medium-, and high-risk groups according to ascending mean risk scores.

For proteomic predictors, we built multiple regularized Cox models. Ridge-Cox and Lasso-Cox models were fitted using the *glmnet* R package (v4.1-8). Spike-and-slab lasso (sslasso) Cox models [82] were implemented using *BhGLM* (v1.1.0) with a fixed slab scale (*s*_1_ = 0.5) and a sequence of 25 spike scales (*s*_0_ from 0.0001 to 0.49, step = 0.02). The optimal model was selected via 10-fold cross-validation based on deviance. Gradient boosting models (glmboost) [68] were fitted using the *mboost* package (v2.9-10) with a learning rate of 0.1, and the optimal number of iterations (*m_stop_*) was determined by the *cvrisk* function. All four modeling strategies were systematically applied under identical settings to nine distinct predictor sets: the eight protein expression mode-defined sets (SU, SD, US, UD, UU, DU, DS, and DD) and their union (“All”).

Model performance was evaluated using the concordance index (C-index) [83] and time-dependent area under the ROC curve (tdAUC) [84], both computed with the *survcomp* package (v1.54.0). Each metric ranges from 0 to 1, with higher values indicating superior predictive accuracy. Time-dependent AUCs were plotted from 1 to 7 years to provide a comprehensive temporal comparison across models.

Key Points

- Proteomic profiling of the Healthy–NAT–Tumor continuum in ESCC defines NAT as a molecularly intermediate tissue.
- NAT-derived proteomic and immune subtypes improve patient stratification and prognostic prediction.
- Dynamic protein expression modes uncover early carcinogenesis events and a US-protein-based prognostic signature.
- Novel biomarkers and targets, including complement proteins and mesenchymal stem cells, offer translational potential for ESCC.

## Supplementary Material

bbag186_Supplementary_material

## Data Availability

The proteome data of 124 paired NAT and tumor samples and phosphoproteome data used in this study were obtained from previously published datasets [7]. All other data supporting the findings of this study are available within the paper and its Supplementary Information files.

## Code availability

All custom R scripts used for data processing, statistical analysis, and figure generation in this study are publicly available on GitHub at https://github.com/weiliu123/ESCC-proteomics-analysis. The repository includes annotated scripts and a README file describing the analysis workflow and software dependencies.
