# Supplementary material for: Proteomic analysis across Healthy–NAT–Tumor tissues uncovers clinically relevant biological events in esophageal squamous cell carcinoma
Source: Brief Bioinform. 2026 Apr 24;27(2):bbag186. doi: 10.1093/bib/bbag186 (PMC13107185; doi:10.1093/bib/bbag186)
Supplement: bbag186_Supplementary_material [file bbag186_supplementary_material.zip › Supplementary Methods.docx]

## Supplementary Methods

**LC-MS/MS Analysis**

Proteomic analysis of the 20 Healthy samples was performed alongside the previously reported 124 paired NAT-Tumor samples using an identical TMT-based quantitative workflow [1]. Specifically, the same internal reference—a pooled mixture of 60 randomly selected NAT-Tumor pairs from the prior study—was used. Healthy tissues were processed by the Filter Aided Sample Preparation (FASP) method. The resulting tryptic peptides were desalted using StageTips, lyophilized, and labeled with TMT-11plex reagent (Pierce) as per the manufacturer's protocol.

The 20 Healthy samples were analyzed over two TMT-11plex experiments. In each experiment, the internal reference was labeled with the 131C channel, and ten individual samples were assigned to the other ten TMT channels. For each experiment, 200 μg of labeled peptides were fractionated by basic reversed-phase chromatography (bRP) on an Ultimate 3000 HPLC system equipped with a Waters XBridge BEH C18 column (5 μm, 4.6 × 250 mm). Mobile phase A contained 0.1% formic acid (FA, v/v) in water; mobile phase B contained 0.1% FA in 80% acetonitrile (ACN). A linear gradient from 5% to 40% mobile phase B over 90 minutes, followed by a rise to 70% B in 6 minutes at 1 mL/min, was applied. The collected 96 fractions were consolidated into 32 final fractions and lyophilized.

LC-MS/MS data acquisition was performed on an Orbitrap Fusion Lumos mass spectrometer fitted with a nanoelectrospray source and coupled to either an EASY-nLC 1200 or a nanoU3000 UHPLC system (Thermo Fisher Scientific). Peptides were separated on a reversed-phase analytical column (75 μm i.d. × 25 cm, packed with 2 μm C18 beads) using a 120-minute gradient from 9% to 32% ACN (in 0.1% FA), then to 50% B in 20 minutes, at a flow rate of 300 nL/min.

Mass spectrometry settings were as follows: The spray voltage was set at 2.2 kV, and the ion transfer capillary temperature was maintained at 300 °C. Full-scan MS spectra (m/z 350–1500) were acquired at 60,000 resolution with an AGC target of 4×10^5^ and a maximum injection time of 50 ms. The top ions were selected for HCD-MS2 fragmentation with 38% normalized collision energy in a 3-second cycle. MS2 spectra were collected at 50,000 resolution with an AGC target of 1×10^5^ and a maximum injection time of 105 ms, using a 0.7 m/z isolation window. Dynamic exclusion was set to 30 seconds, and ions with unassigned charge states or charges of 1+ and >7+ were excluded from fragmentation.

The MS data were collected using *Xcalibur* software (Thermo Fisher Scientific, version 3.0). Raw data were processed with Proteome Discoverer (v. 2.2) and searched against the SwissProt human database. Peptide and protein identifications were filtered to achieve a false discovery rate (FDR) of less than 1%, requiring at least one unique peptide per protein.

**Global proteomic data analysis**

For comparative analysis across tissue types, principal component analysis (PCA) and hierarchical clustering were performed in R (v.4.4.0) using the 5,386 proteins consistently quantified in all 20 Healthy, 124 NAT, and 124 Tumor samples. Confidence intervals for the PCA were calculated using the *ggbiplot* R package (v.0.6.2).

Differential expression analysis was conducted using non-parametric tests. The Wilcoxon rank-sum test was applied for unpaired comparisons (NAT vs. Healthy; Tumor vs. Healthy), while the Wilcoxon signed-rank test was used for the paired comparison (Tumor vs. NAT). Proteins with a Benjamini-Hochberg (BH) adjusted *p*-value < 0.01 and an absolute fold change > 1.5 were defined as differentially expressed.

**NAT proteomic subtype analysis**

NAT proteomic subtypes were identified by consensus clustering performed with the R package *ConsensusClusterPlus* (v.1.50.0) [2]. The analysis was based on the top 25% most variable proteins, ranked by coefficient of variation. Key parameters were: maxK = 6, reps = 1000, pItem = 0.8, pFeature = 0.8, clusterAlg = "hc", and distance = "spearman". The optimal number of clusters (*k*=2) was determined by maximizing the average silhouette width, computed using the *cluster* R package (v.2.1.0).

Differential expression and phosphorylation between the NAT2 and NAT1 subtypes were assessed using the Wilcoxon rank-sum test. For proteomic data, proteins with a Benjamini-Hochberg (BH) adjusted *p*-value < 0.01 and an absolute fold change > 1.2 were deemed significant (**Table S2C**). For phosphoproteomic data, phosphosites with a *p*-value < 0.01 and a fold change > 2 or < 0.5 were considered significantly dysregulated (**Table S2F**).

**Tissue composition analysis**

Tissue cellular composition was deconvoluted from proteomic data using xCell [3], a method based on single-sample Gene Set Enrichment Analysis (ssGSEA) [4] that has been applied to proteomic datasets [5-7]. After removing dependencies between closely related cell types, we estimated the abundances of 30 immune and stroma cell types that are assumed to reside in the tumor and tissue microenvironment using the *xCell* R package (v.1.1.0) [3].

To identify clinically relevant immune subtypes, we performed consensus clustering on the xCell enrichment scores using the following parameters: maxK = 6, reps = 1000, pItem = 0.8, pFeature = 1, clusterAlg = "km", and distance = "euclidean". Survival differences between the resulting immune subtypes were assessed using the log-rank test, with a *p*-value < 0.05 considered statistically significant.

**Functional enrichment analysis**

Proteomaps were generated via the *Proteomaps* web tool (www.proteomaps.net) to visualize functional categories enriched among proteins upregulated in NATs or Tumors [8]. Two-dimensional annotation enrichment was conducted with *Perseus* (v.2.0.11) [9, 10]. Pathway enrichment analysis was performed using either *Metascape* [11] or the R package *clusterProfiler* (v.4.12.0) [12], with statistical significance assessed by the hypergeometric test followed by Benjamini-Hochberg (BH) correction; an adjusted *p*-value (q-value) < 0.05 was considered significant.

PTM-SEA was executed with ssGSEA2.0 (<https://github.com/broadinstitute/ssGSEA2.0>) against the PTMsigDB (v1.9.0; <https://proteomics.broadapps.org/ptmsigdb/>) to identify enriched post-translational modification signatures [13]. Differences in signature enrichment scores between NAT2 and NAT1 subtypes were evaluated using an unpaired two-sided Student's t-test, with signatures yielding a *p*-value < 0.05 deemed significantly dysregulated.

Gene Set Variation Analysis (GSVA) was applied using the R package *GSVA* (v.1.52.3) [14] to calculate enrichment scores for the TASA gene set, the eight protein expression mode sets, and the hallmark gene sets [55]. The GSVA enrichment scores represent the relative activity of each gene set in a given sample, where a higher score corresponds to higher activity.

**Survival analysis**

OS and DFS differences between groups were assessed using Kaplan-Meier survival curves, and the statistical significance was determined by the log-rank test, implemented with the R package *survminer* (v.0.4.9). Univariate and multivariate Cox proportional hazards regression analyses were employed to evaluate the association between clinicopathologic variables and patient survival (OS/DFS). Variables yielding a *p*-value < 0.05 in the univariate analysis were included in the subsequent multivariate model. Factors that maintained a p-value < 0.05 in the multivariate analysis were considered independent prognostic factors. Associations between clinicopathologic factors and the NAT proteomic or immune subtypes were tested using the χ²-test or Fisher's exact test for categorical variables, and the Wilcoxon rank-sum test for continuous variables.

**Prognostic model construction and evaluation**

We constructed two primary models based on NAT proteomic subtypes: 1) The "NAT subtype+pTNM" model, a Cox PH model integrating the NAT proteomic subtype and pTNM stage; 2) The "NAT subtype+pTNM 3c" staging system, derived by applying k-means clustering (k=3) to the risk scores from the first model. The resulting three patient clusters were designated as low-, medium-, and high-risk based on ascending average risk scores.

For proteomic predictors, we built multiple regularized Cox models. Ridge-Cox and Lasso-Cox models were fitted using the *glmnet* R package (v.4.1-8). The spike-and-slab lasso (sslasso) Cox models [15] were implemented with the *BhGLM* package (v.1.1.0), employing a fixed slab scale (*s*_1_ = 0.5) and a sequence of 25 spike scales (*s*_0_ from 0.0001 to 0.49, step=0.02), with the optimal model selected via 10-fold cross-validation based on deviance. Gradient boosting models (glmboost) [16] were fitted using the *mboost* package (v.2.9-10) with a learning rate of 0.1, where the optimal number of iterations (*m_stop_*) was determined by the *cvrisk* function. These approaches were applied to nine distinct predictor sets: the eight individual protein expression mode sets (SU, SD, etc.) and their union ("All").

Model performance was evaluated using the concordance index (C-index) [17] and time-dependent area under the ROC curve (tdAUC) [18], computed with the *survcomp* package (v.1.54.0). Both metrics range from 0 to 1, with higher values indicating superior predictive accuracy. The tdAUC, which assesses performance at a specific time point, was plotted from 1 to 7 years to provide a comprehensive temporal comparison of the models.

**References**

1. Liu, W., et al., *Large-scale and high-resolution mass spectrometry-based proteomics profiling defines molecular subtypes of esophageal cancer for therapeutic targeting.* Nature Communications, 2021. **12**(1): p. 4961.

2. Wilkerson, M.D. and D.N. Hayes, *ConsensusClusterPlus: a class discovery tool with confidence assessments and item tracking.* Bioinformatics, 2010. **26**(12): p. 1572-1573.

3. Aran, D., Z. Hu, and A.J. Butte, *xCell: digitally portraying the tissue cellular heterogeneity landscape.* Genome Biology, 2017. **18**(1): p. 220.

4. Barbie, D.A., et al., *Systematic RNA interference reveals that oncogenic KRAS-driven cancers require TBK1.* Nature, 2009. **462**(7269): p. 108-112.

5. Clark, D.J., et al., *Integrated Proteogenomic Characterization of Clear Cell Renal Cell Carcinoma.* Cell, 2019. **179**(4): p. 964-983.e31.

6. Petralia, F., et al., *Integrated Proteogenomic Characterization across Major Histological Types of Pediatric Brain Cancer.* Cell, 2020. **183**(7): p. 1962-1985.e31.

7. Xu, N., et al., *Integrated proteogenomic characterization of urothelial carcinoma of the bladder.* Journal of Hematology & Oncology, 2022. **15**(1): p. 76.

8. Liebermeister, W., et al., *Visual account of protein investment in cellular functions.* Proceedings of the National Academy of Sciences, 2014. **111**(23): p. 8488-8493.

9. Tyanova, S., et al., *The Perseus computational platform for comprehensive analysis of (prote)omics data.* Nature Methods, 2016. **13**(9): p. 731-740.

10. Cox, J. and M. Mann, *1D and 2D annotation enrichment: a statistical method integrating quantitative proteomics with complementary high-throughput data.* BMC Bioinformatics, 2012. **13**(16): p. S12.

11. Zhou, Y., et al., *Metascape provides a biologist-oriented resource for the analysis of systems-level datasets.* Nature Communications, 2019. **10**(1): p. 1523.

12. Yu, G., et al., *clusterProfiler: an R Package for Comparing Biological Themes Among Gene Clusters.* OMICS: A Journal of Integrative Biology, 2012. **16**(5): p. 284-287.

13. Krug, K., et al., *A Curated Resource for Phosphosite-specific Signature Analysis.* Molecular & Cellular Proteomics, 2019. **18**(3): p. 576-593.

14. Hänzelmann, S., R. Castelo, and J. Guinney, *GSVA: gene set variation analysis for microarray and RNA-Seq data.* BMC Bioinformatics, 2013. **14**(1): p. 7.

15. Tang, Z., et al., *The spike-and-slab lasso Cox model for survival prediction and associated genes detection.* Bioinformatics, 2017. **33**(18): p. 2799-2807.

16. Bühlmann, P. and T. Hothorn, *Boosting Algorithms: Regularization, Prediction and Model Fitting.* Statistical Science, 2007. **22**(4): p. 477-505, 29.

17. HARRELL Jr., F.E., K.L. LEE, and D.B. MARK, *Multivariable prognostic models: issues in developing models, evaluating assumptions and adequacy, and measuring and reducing errors.* Statistics in Medicine, 1996. **15**(4): p. 361-387.

18. Heagerty, P.J., T. Lumley, and M.S. Pepe, *Time-Dependent ROC Curves for Censored Survival Data and a Diagnostic Marker.* Biometrics, 2004. **56**(2): p. 337-344.
