# Supplementary material for: Proteomic analysis across Healthy–NAT–Tumor tissues uncovers clinically relevant biological events in esophageal squamous cell carcinoma
Source: Brief Bioinform. 2026 Apr 24;27(2):bbag186. doi: 10.1093/bib/bbag186 (PMC13107185; doi:10.1093/bib/bbag186)
Supplement: bbag186_Supplementary_material [file bbag186_supplementary_material.zip › Supplementary Figures and Tables.20260330.docx]

**Proteomic analysis across Healthy–NAT–Tumor tissues uncovers clinically relevant biological events in esophageal squamous cell carcinoma**

Wei Liu^1,#,*^, Wei Wang^1,#^, Dan-Wei Zheng^2,#^, Ying-Qin Ran^2^, Ming-Xiao Feng^2^, Dan-Xia Deng^2^, Xiu-E Xu^2^, Li-Yan Xu^2^, Hai-Hua Huang^2*^, En-Min Li^2,3,4*^

**Supplementary Figures**

**
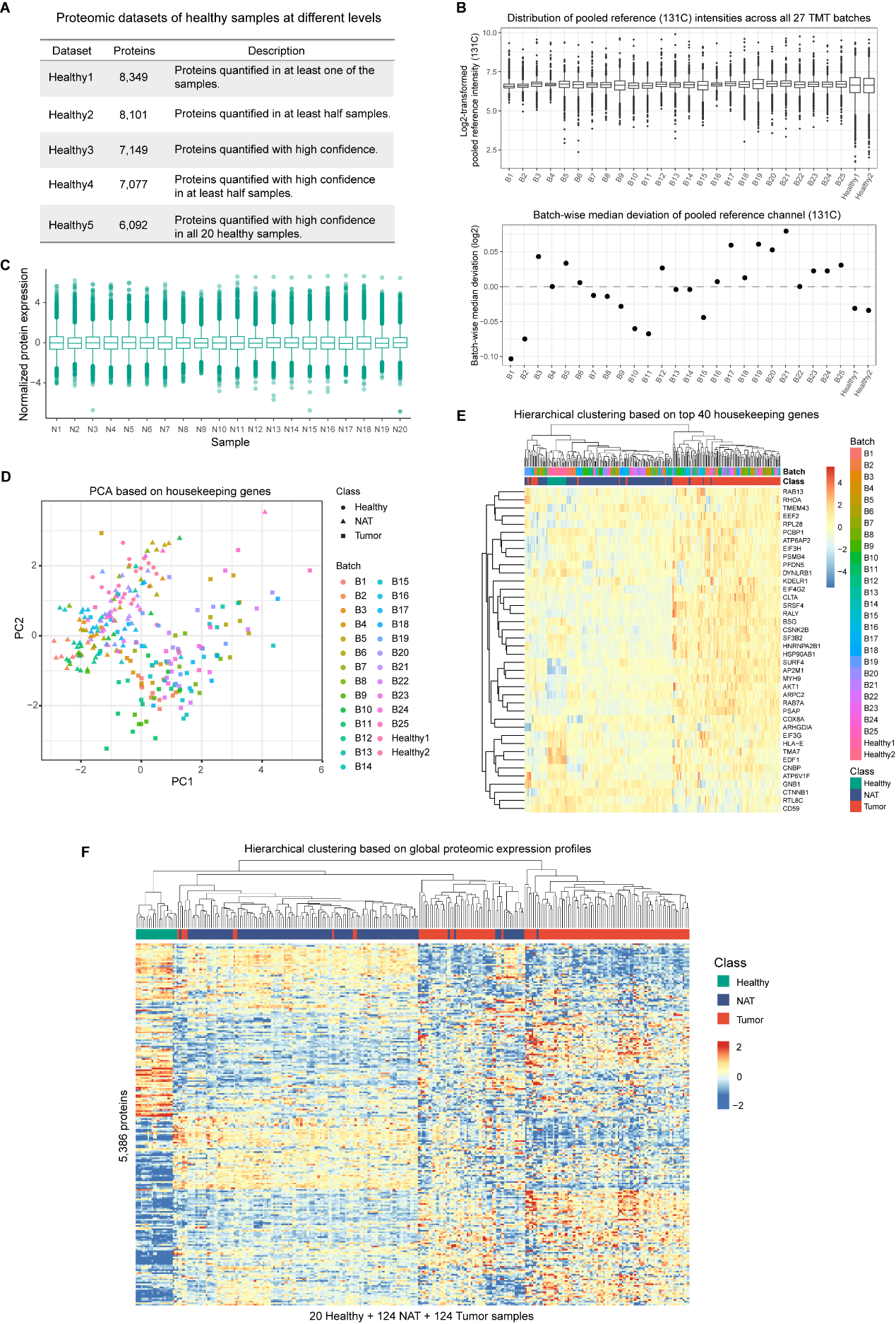
**

**Figure S1. Quality assessment of TMT-based proteomic data from Healthy samples.**

(**A**) Numbers of proteins identified at different confidence levels.

(**B**) Assessment of cross-batch consistency using the pooled internal reference (131C).

Top: Boxplots showing the distribution of log_2_-transformed reference intensities (log_2_[reference + 1]) for proteins quantified in all 27 TMT batches, including 25 batches for paired NAT–Tumor samples and 2 batches for Healthy samples. Bottom: Batch-wise median deviation of the reference channel relative to the global median, shown on the log_2_ scale.

(**C**) Distribution of protein expression values for 7,077 quantified proteins in the Healthy4 dataset.

(**D**) Principal component analysis (PCA) based on housekeeping genes. PCA was performed using the top 40 housekeeping genes derived from the intersection of mucosa-, muscularis-, and gastroesophageal junction–specific reference genes curated from the Housekeeping and Reference Transcript Atlas (HRT Atlas v1.0). Each point represents one sample, colored by tissue type. This analysis was used to evaluate whether samples cluster primarily by tissue type rather than by batch.

(**E**) Hierarchical clustering of samples based on housekeeping gene expression. Heatmap showing the expression patterns of the top 40 housekeeping genes across Healthy, NAT, and Tumor samples. Clustering was performed using Euclidean distance and complete linkage.

(**F**) Hierarchical clustering of Healthy, NAT, and Tumor samples based on global proteomic profiles. Hierarchical clustering was performed using protein expression levels of 5,386 proteins quantified across all samples (20 Healthy, 124 NAT, and 124 Tumor). Euclidean distance was used to calculate sample-to-sample dissimilarity, and complete linkage was applied for clustering. The heatmap displays scaled protein expression values, illustrating global proteomic similarities and differences among sample types.
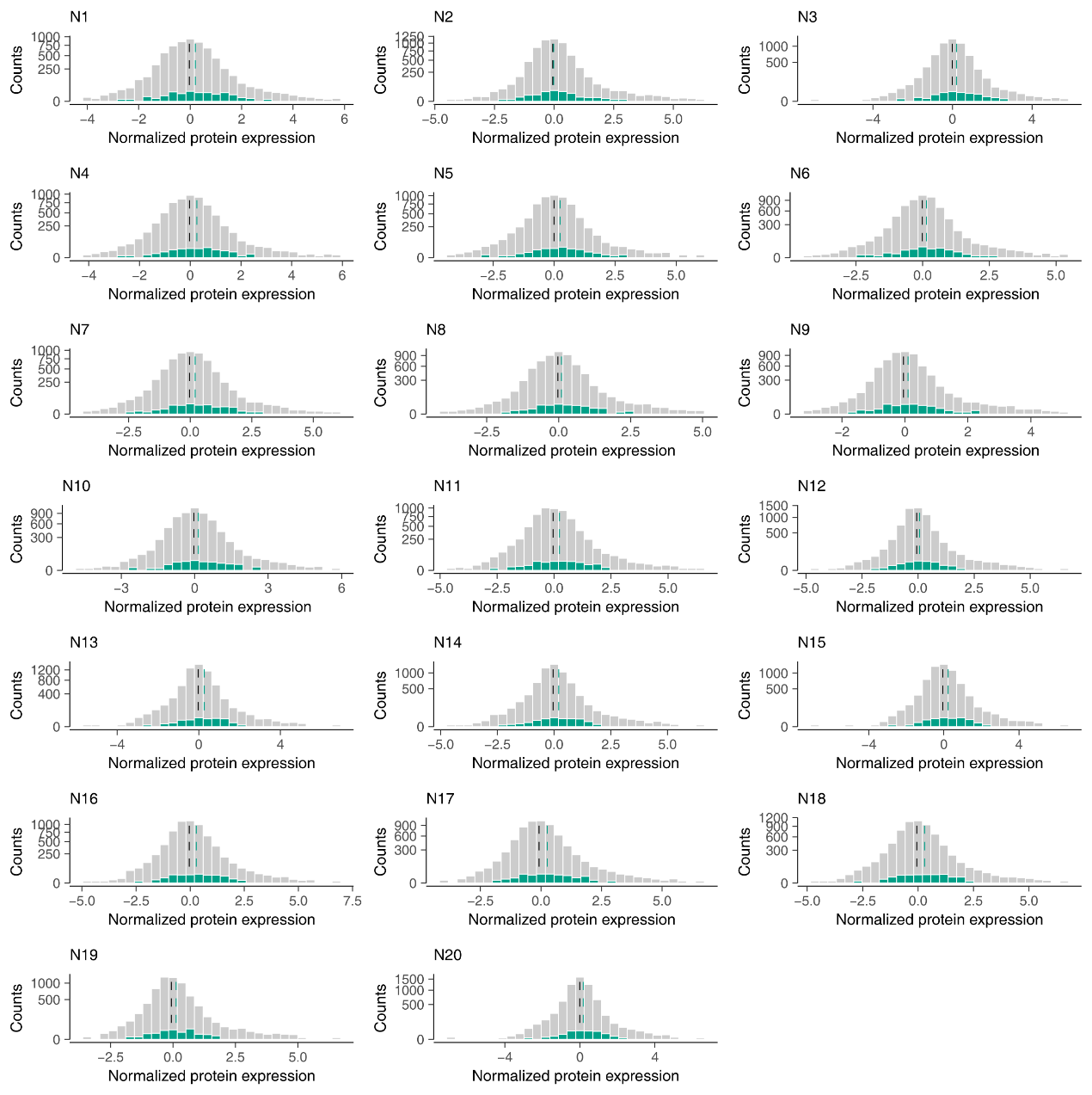


**Figure S2. Expression distribution of all proteins and esophagus-specific proteins in individual Healthy samples.**


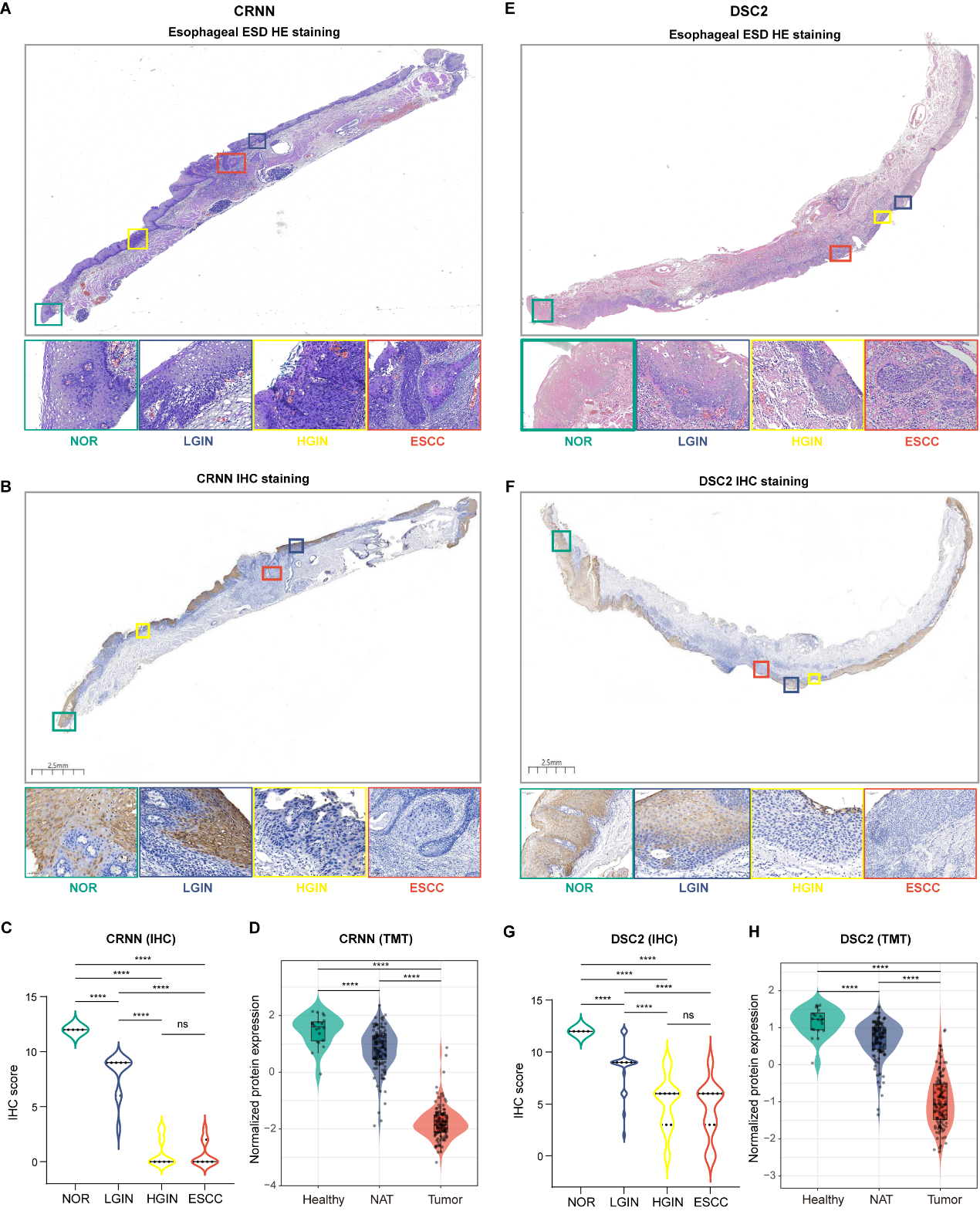


**Figure S3. CRNN and DSC2 expression in esophageal lesions and immunohistochemical validation.**

(**A**) Hematoxylin and eosin (H&E) staining of esophageal endoscopic submucosal dissection (ESD) specimens, shown as whole-slide images and representative magnified regions of interest. Tissue types are annotated as follows: NOR, normal adjacent epithelium; LGIN, low-grade intraepithelial neoplasia; HGIN, high-grade intraepithelial neoplasia; ESCC, esophageal squamous cell carcinoma.

(**B**) Immunohistochemical (IHC) staining of CRNN protein, shown as whole-slide images and representative magnified views.

(**C**) Quantification of CRNN IHC scores across normal and neoplastic esophageal tissues. Statistical significance was assessed using one-way analysis of variance (ANOVA).

(**D**) Violin plot showing normalized CRNN protein abundance in Healthy, NAT, and Tumor samples quantified by TMT-based proteomics. Statistical significance between Healthy vs. NAT and Healthy vs. Tumor was assessed using the Wilcoxon rank-sum test, while the NAT vs. Tumor comparison was performed using the Wilcoxon signed-rank test.

(**E**–**H**) Corresponding analyses for DSC2, including IHC staining, IHC score quantification across esophageal lesions, and TMT-based proteomic quantification, presented in the same manner as in panels (**B**–**D**).


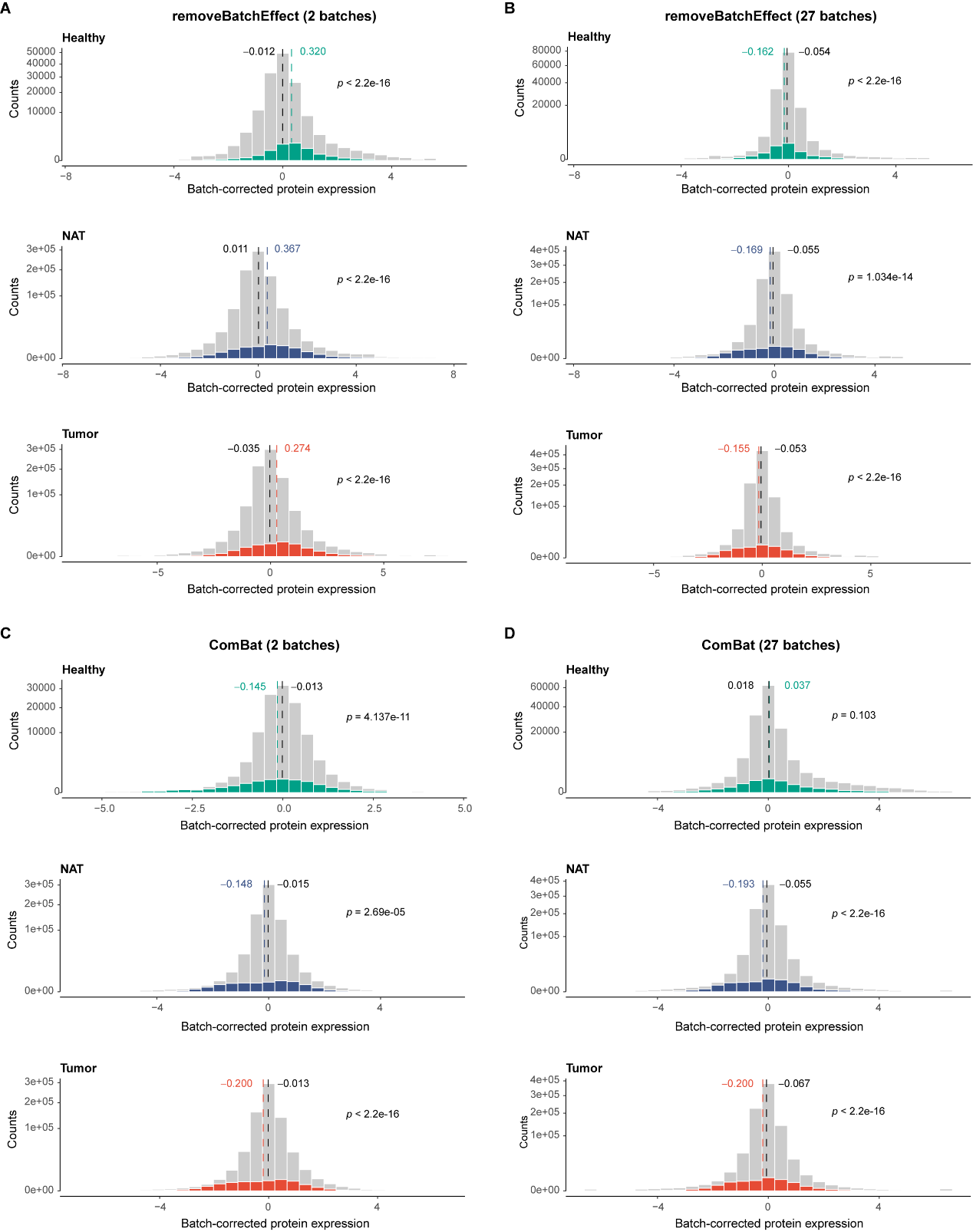


**Figure S4. Effect of different batch correction strategies on protein expression distributions.** Distribution of batch-corrected expression values for all proteins (gray) and esophagus-specific proteins (colored) across sample classes (Healthy, NAT, and Tumor) after batch correction using different methods and batch definitions. (**A**) removeBatchEffect with two batches (Healthy vs. NAT/Tumor). (**B**) removeBatchEffect with 27 batches (individual TMT batches). (**C**) ComBat with two batches (Healthy vs. NAT/Tumor). (**D**) ComBat with 27 batches (individual TMT batches). Median expression values are indicated by dashed vertical lines (black for all proteins and colored for esophagus-specific proteins). Statistical significance between all proteins and esophagus-specific proteins was assessed using a two-sided Wilcoxon rank-sum test, and corresponding *p* values are shown in each panel.


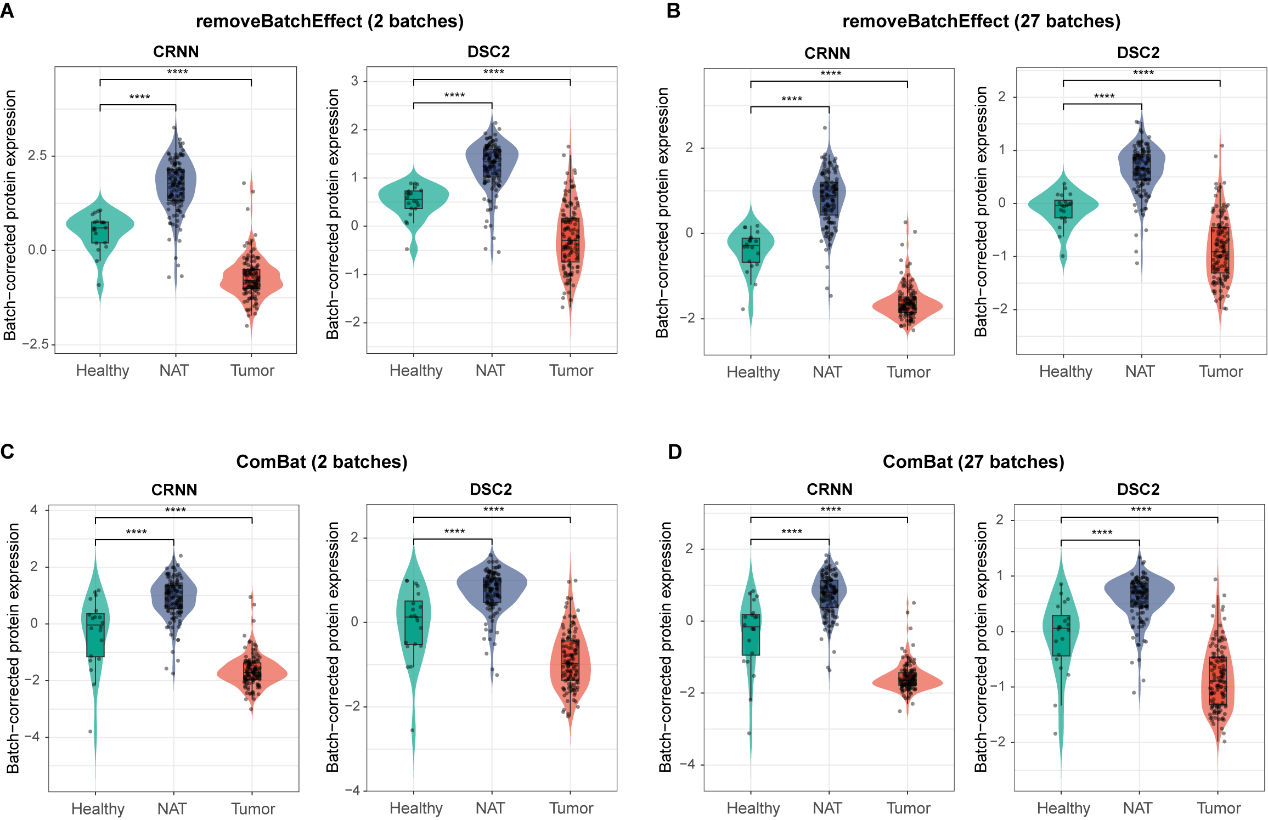


**Figure S5. Computational batch correction leads to over-correction inconsistent with IHC validation.** Violin plots showing batch-corrected expression levels of CRNN and DSC2 across Healthy, NAT, and Tumor samples following different batch correction strategies: (**A**) removeBatchEffect with two batches (Healthy vs. NAT/Tumor); (**B**) removeBatchEffect with 27 batches (individual TMT batches); (**C**) ComBat with two batches; (**D**) ComBat with 27 batches.


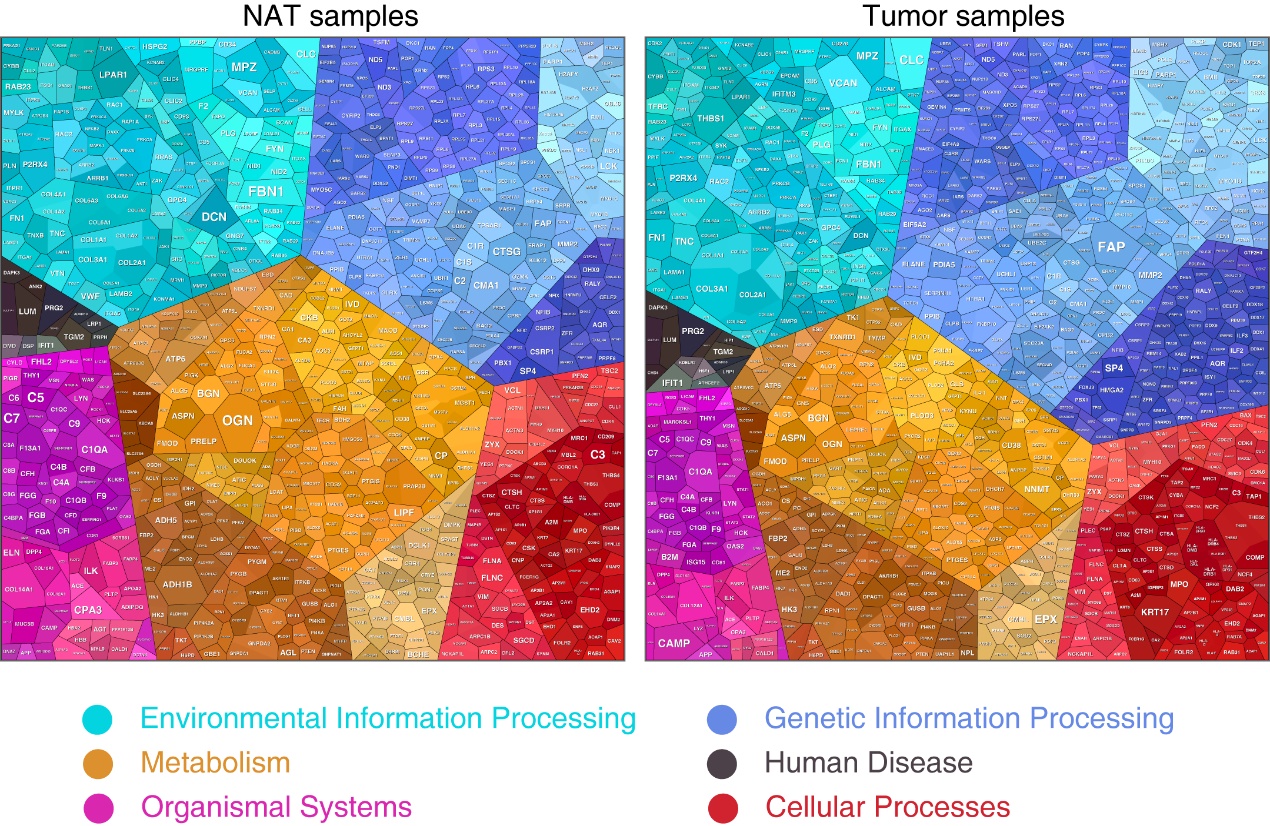


**Figure S6. Functional categories elevated in NATs and Tumors compared with Healthy samples.**

Left: NATs; Right: Tumors. Each polygon represents a single protein significantly different by the two-sided Wilcoxon rank-sum test. Polygon size correlates with the protein’s fold change.


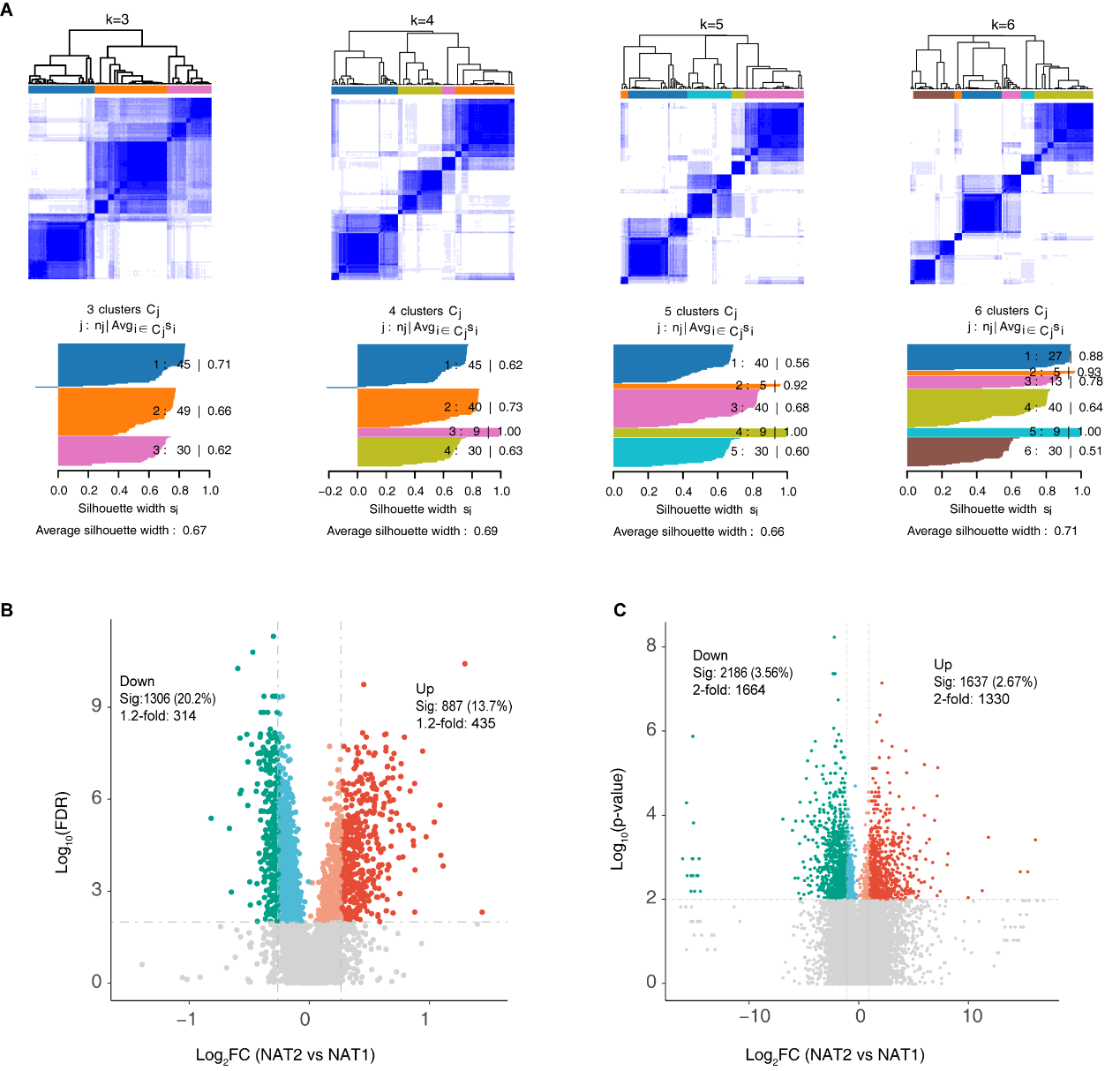


**Figure S7. Proteomic classification of ESCC NAT subtypes.**

(**A**) Consensus clustering of 124 ESCC NAT samples with cluster numbers (*k*) ranging from 3 to 6. The upper panel shows consensus matrices; the lower panel displays silhouette-width plots.

(**B**) Volcano plot of proteins up- and downregulated in subtype NAT2. Light red and green denote proteins with BH-adjusted *p* < 0.01 (significant), whereas red and green indicate proteins with BH-adjusted *p* < 0.01 and fold change > 1.2. Other proteins are shown in gray. *P* values were calculated by two-sided Wilcoxon rank-sum test.

(**C**) Volcano plot of phosphosites up- and downregulated in subtype NAT2. Colors are as in (**B**), except that red and green indicate phosphosites with fold change > 2.


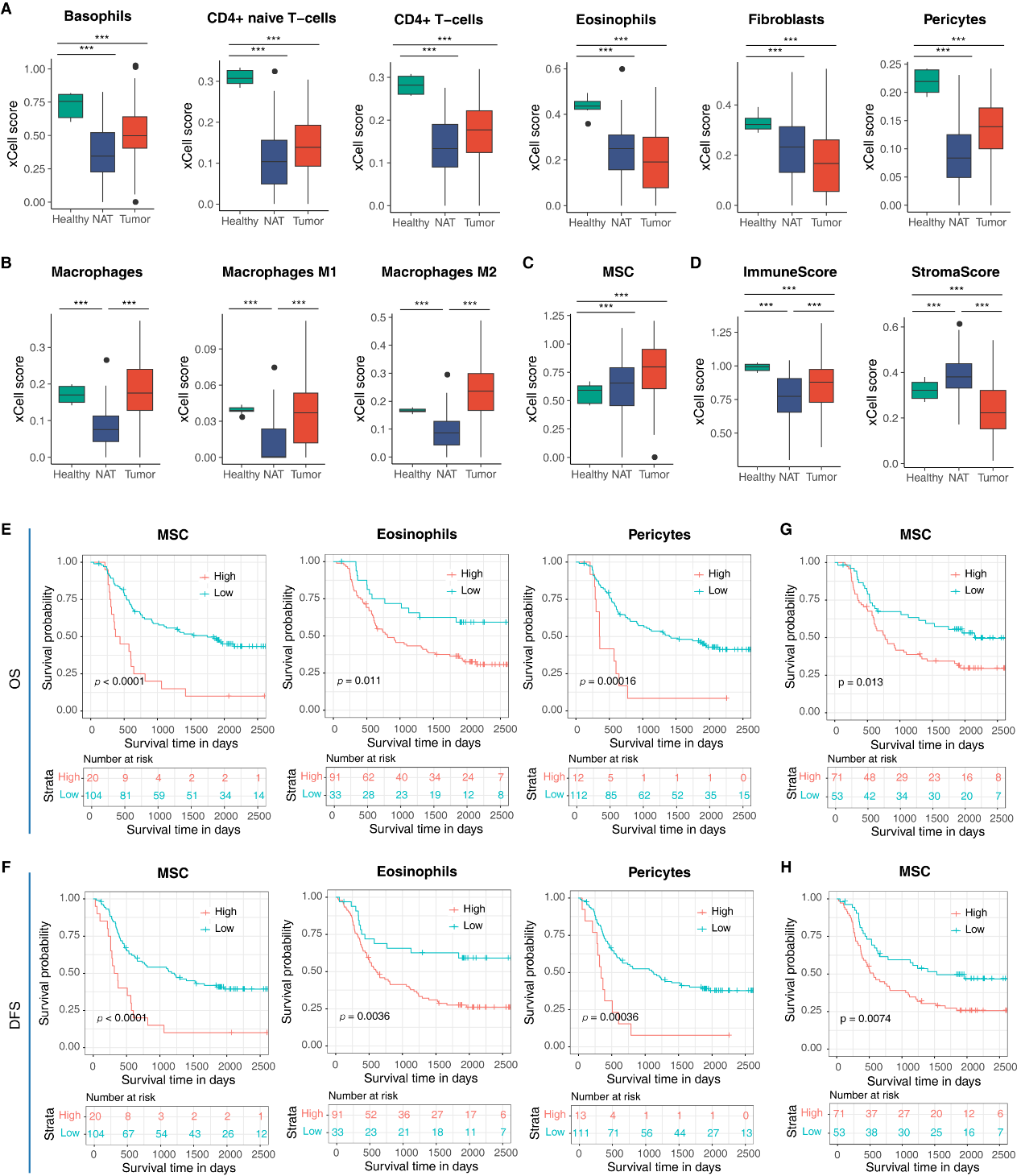


**Figure S8. xCell scores and survival analysis of cell types.**

(**A**–**D**) Boxplots of xCell scores for cell types enriched in Healthy tissues (**A**), depleted in NATs (**B**), and for MSC (**C**) and immune/stromal signatures (**D**). *P* values were determined by two-sided Wilcoxon rank-sum test. *: *p* < 0.05; **: *p* < 0.01; ***: *p* < 0.001.

(**E**–**F**) Kaplan–Meier curves of overall survival (**E**) and disease-free survival (**F**) for MSC, eosinophils, and pericytes in NATs.

(**G**–**H**) Kaplan–Meier curves of overall survival (**G**) and disease-free survival (**H**) for MSC in Tumors. *P* values were determined by two-sided log-rank test.


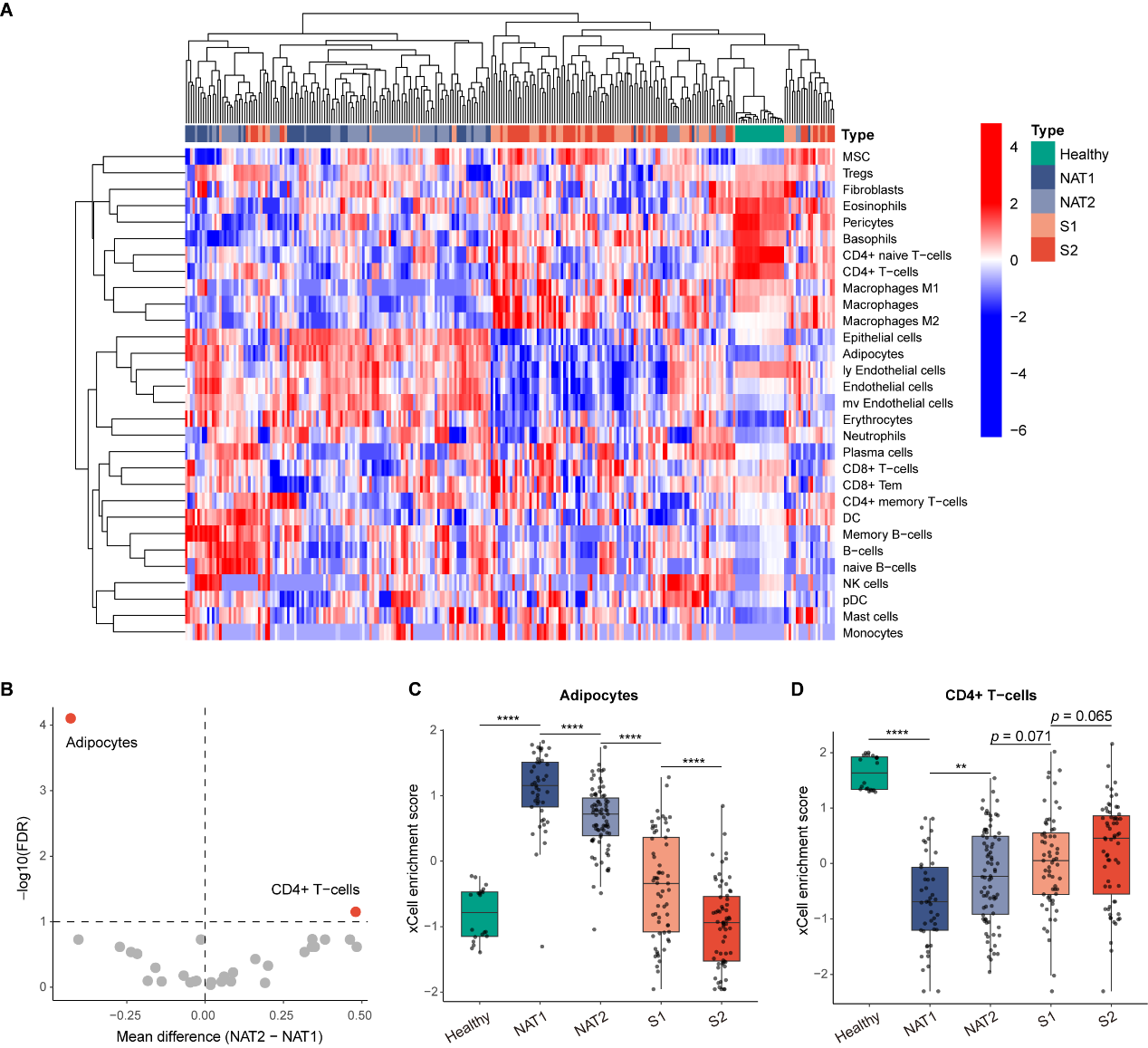


**Figure S9. xCell-based cellular composition differences among proteomic subtypes of NATs and Tumors.**

**(A)** Unsupervised hierarchical clustering of Healthy, NAT, and Tumor samples based on xCell enrichment scores across immune and stromal cell types. Samples include Healthy controls, NAT1 and NAT2 (proteomic subtypes of NAT samples), and S1 and S2 (proteomic subtypes of Tumor samples).

**(B)** Volcano plot showing differential xCell enrichment scores between NAT2 and NAT1 samples. The *x*-axis indicates the mean difference in xCell scores (NAT2 − NAT1), and the *y*-axis represents −log10(FDR-adjusted *P* values). Selected cell types are highlighted.

**(C)** Boxplots of xCell enrichment scores for adipocytes across five sample groups (Healthy, NAT1, NAT2, S1, and S2). Each dot represents an individual sample. Statistical significance was assessed using the one-sided Wilcoxon rank-sum test.

**(D)** Boxplots of xCell enrichment scores for CD4⁺ T cells across the same five sample groups. Statistical testing and data presentation are as in (**C**). FDR-adjusted *p* values are indicated as follows: ****, *p* < 0.0001, **, *p* < 0.01.


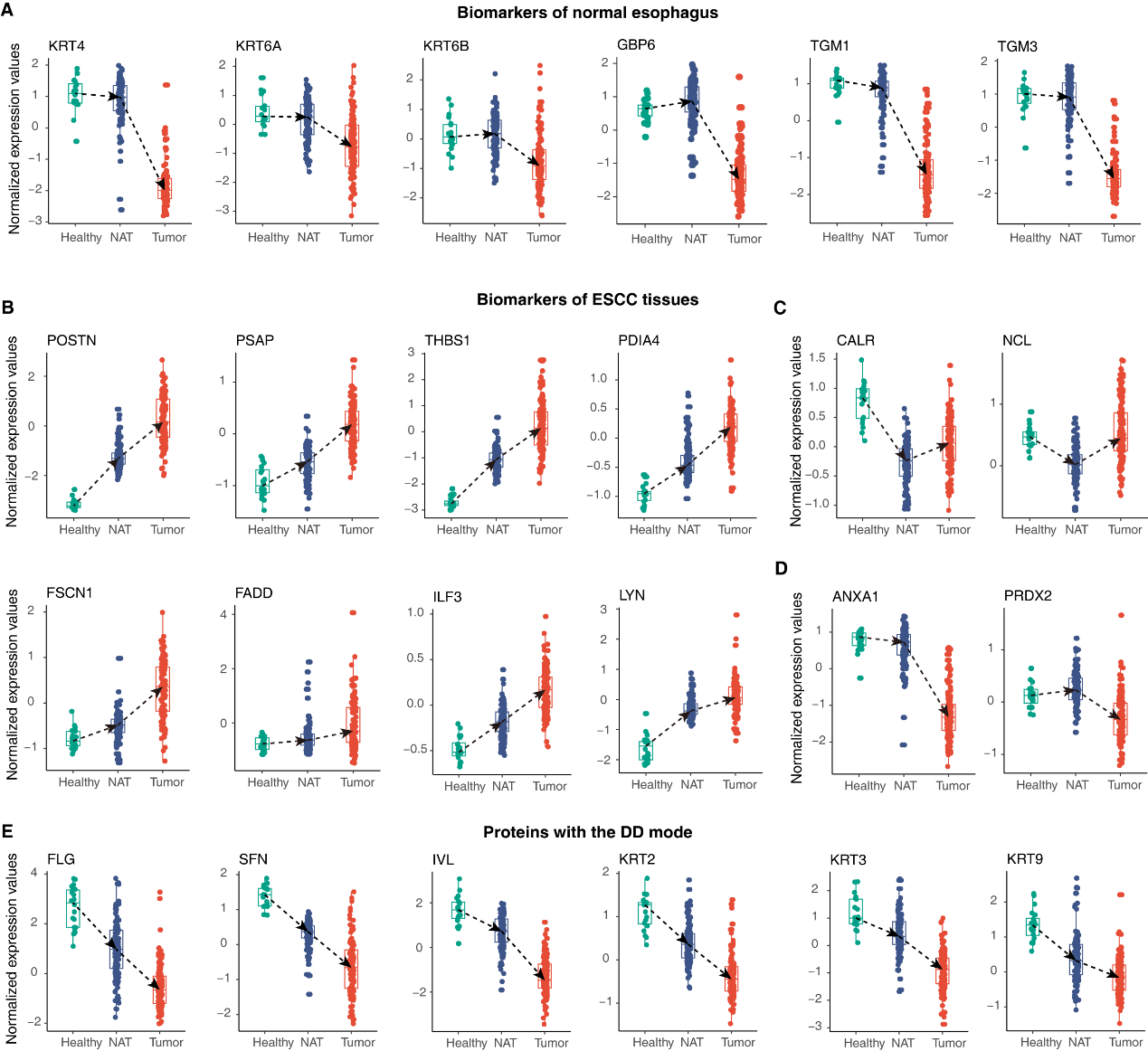


**Figure S10. Boxplots of protein biomarkers.**

(**A**) Biomarkers of normal esophageal tissue.

(**B**–**D**) ESCC biomarkers showing the UU (**B**), DU (**C**), and SD (**D**) expression modes.

(**E**) Proteins following the DD expression mode.


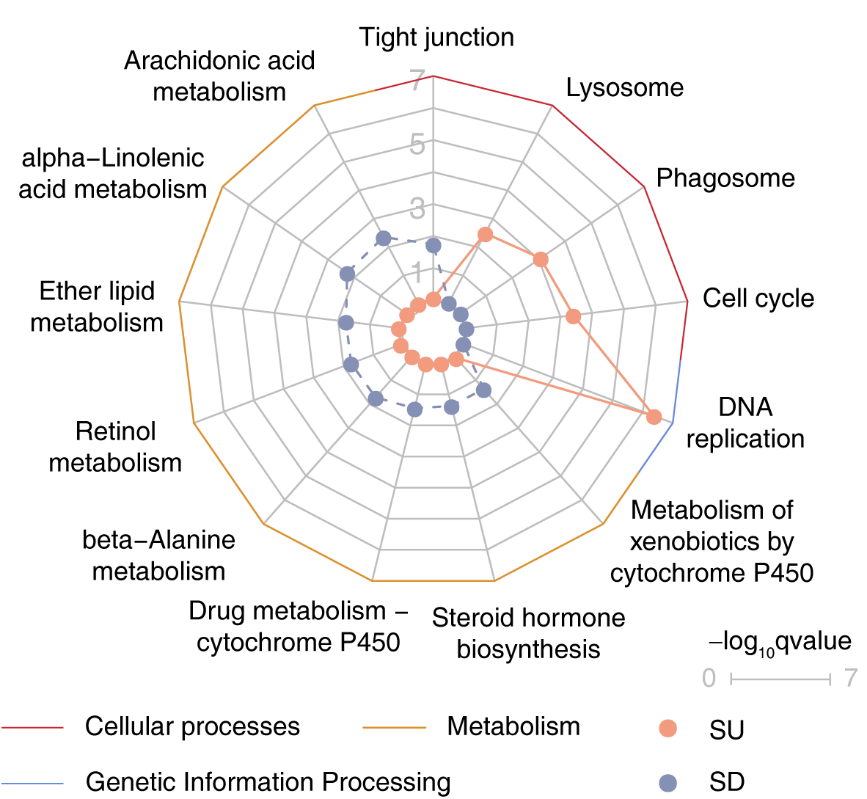


**Figure S11. KEGG pathway enrichment analysis of proteins with SU and SD expression modes.**


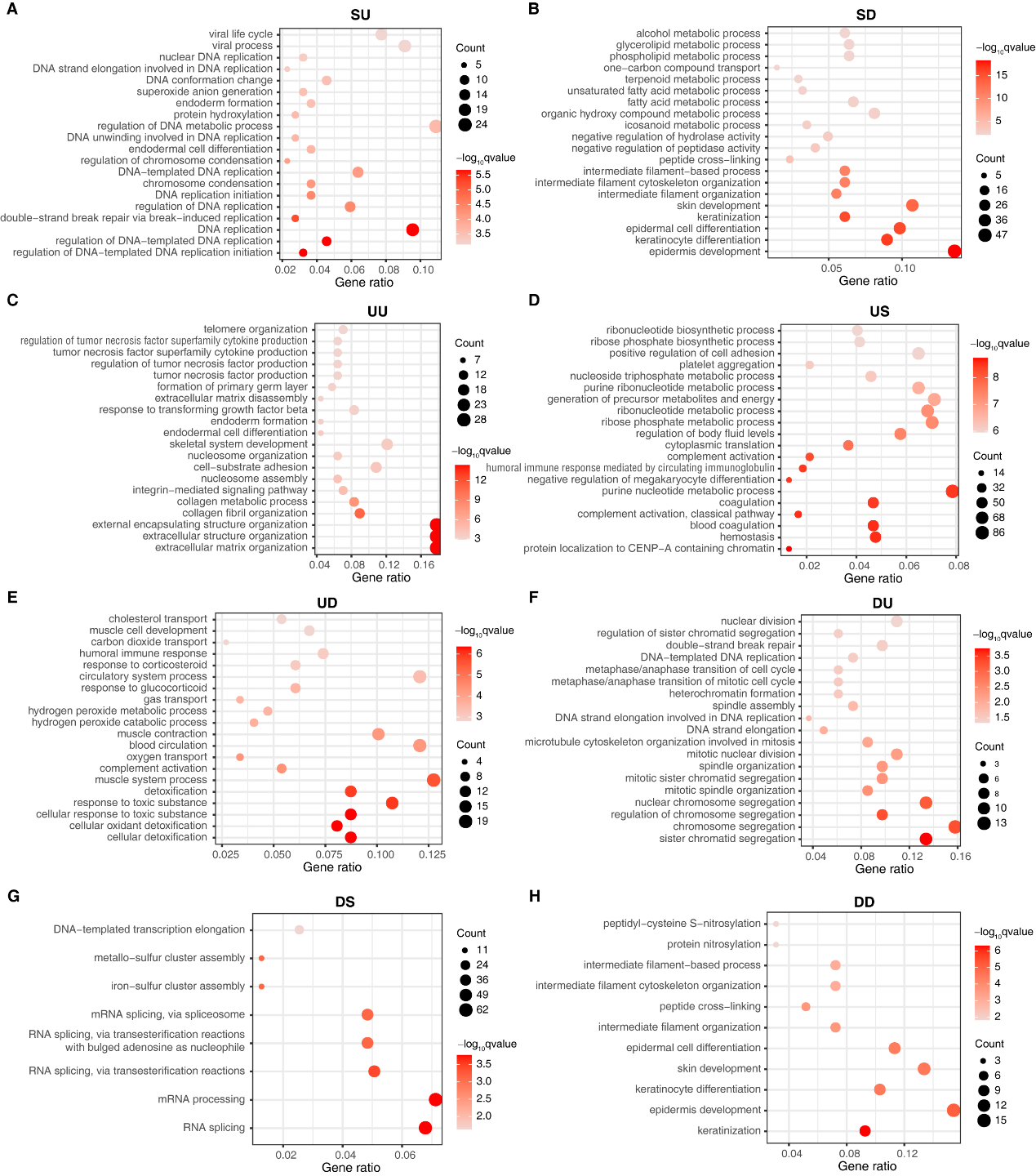


**Figure S12. GO enrichment analysis of proteins grouped by eight expression modes.**

(**A**) SU. (**B**) SD. (**C**) UU. (**D**) US. (**E**) UD. (**F**) DU. (**G**) DS. (**H**) DD.

Top 20 enriched GO biological processes are shown; the full list is provided in **Table S5**.


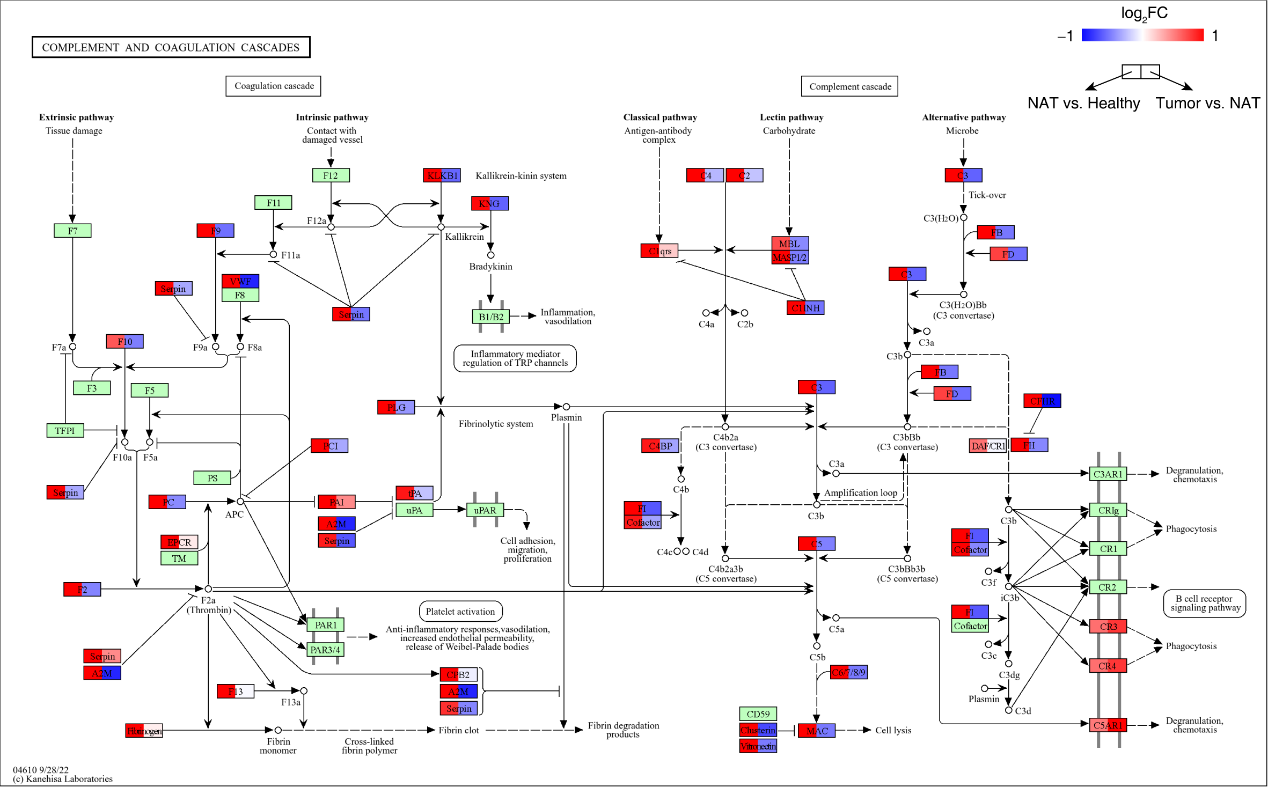


**Figure S13. Dysregulated nodes in the complement and coagulation cascades pathway.**

Fill colors on the left and right sides of each node represent fold changes for NAT vs. Healthy and Tumor vs. NAT, respectively. Red and blue indicate upregulation and downregulation, respectively.


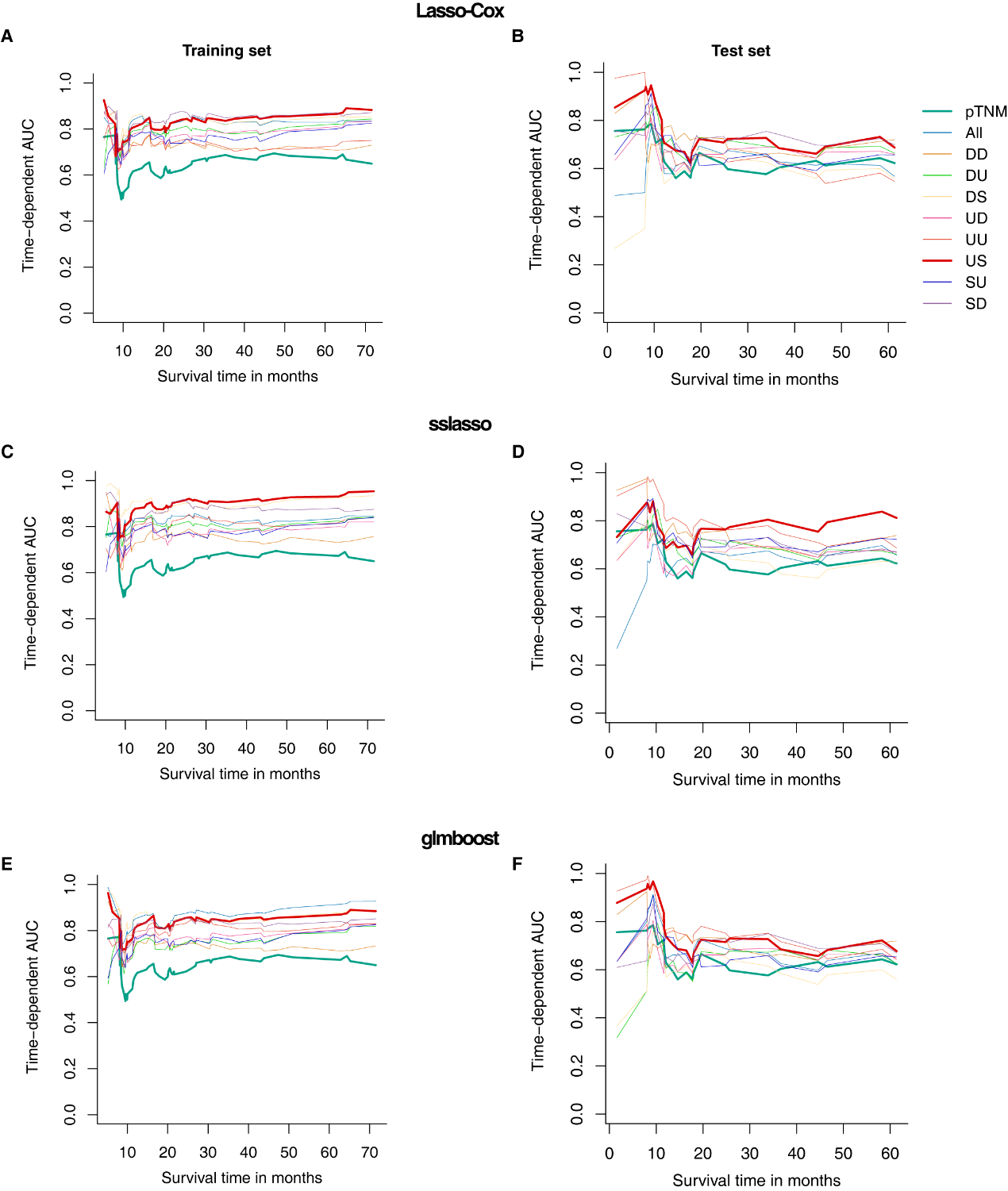


**Figure S14. Prognostic performance of survival models constructed from proteins with eight expression modes.**

(**A**) Lasso-Cox, (**B**) sslasso, and (**C**) glmboost models. Shown are time-dependent AUC curves for overall survival predictions. (**A**, **C**, **E**) Training sets; (**B**, **D**, **F**) test sets.


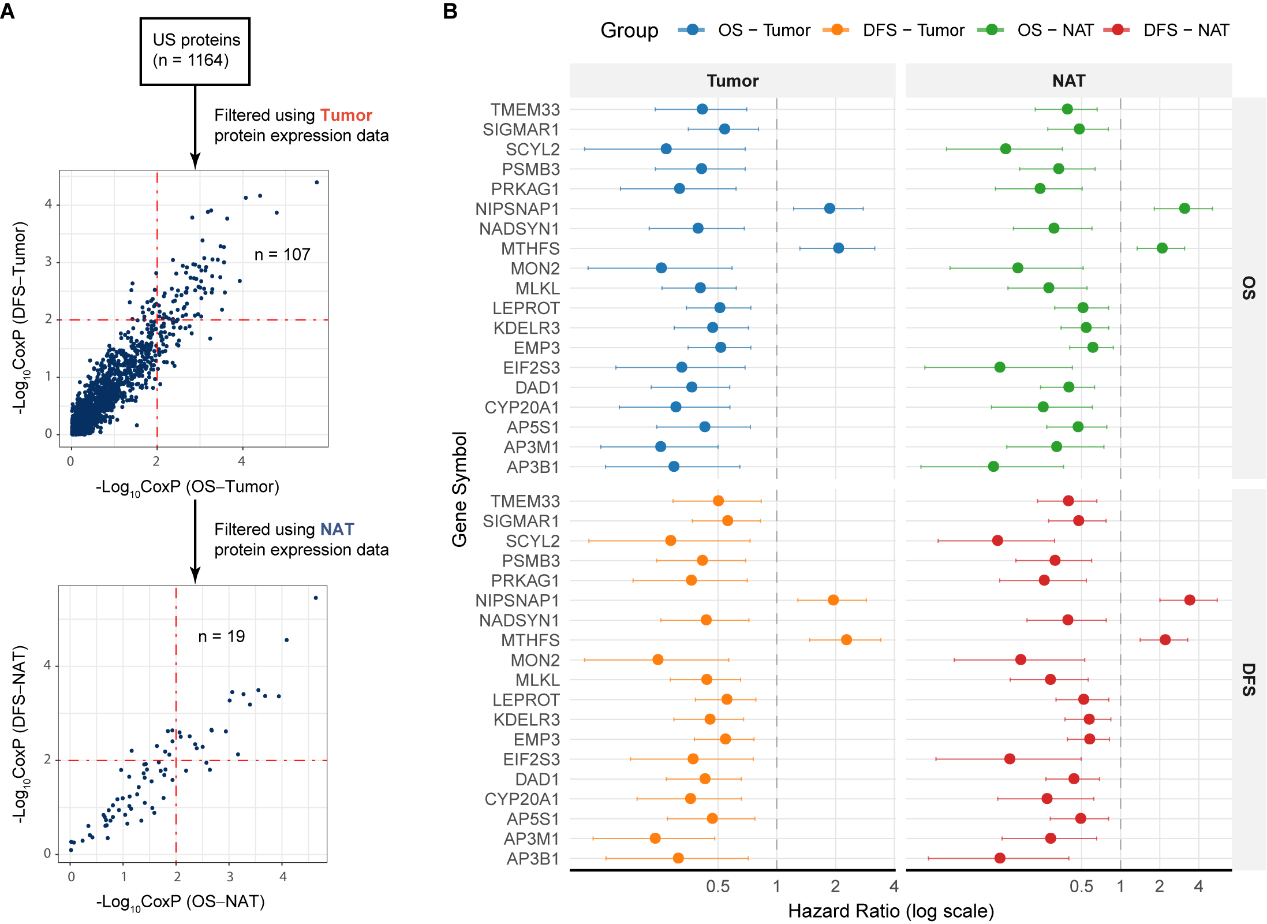


**Figure S15. Identification of prognostic proteins associated with ESCC initiation and progression.**

(**A**) Analytical workflow for identifying proteins significantly associated with both overall survival (OS) and disease-free survival (DFS), based on their expression levels in Tumor (top) and NAT (bottom) samples among the US protein set.

(**B**) Forest plots displaying the hazard ratios and confidence intervals for OS and DFS of the 19 prognostic proteins identified in (**A**), evaluated separately in tumor and NAT samples.
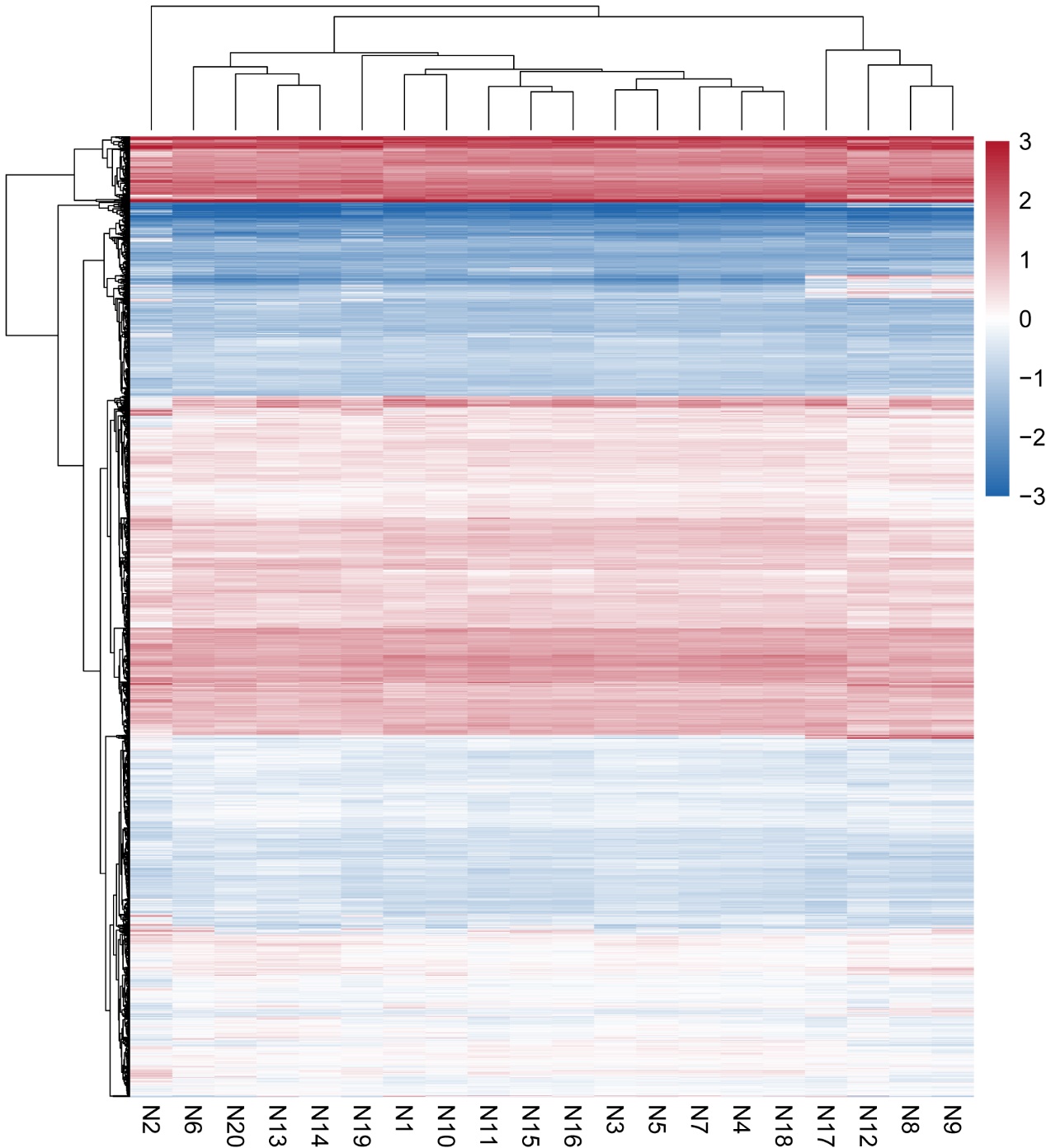


**Figure S16. Hierarchical clustering heatmap of Healthy samples.** Heatmap showing hierarchical clustering of 20 Healthy samples (N1–N20) based on normalized expression values. Columns represent samples and rows represent features. Data were scaled by column prior to visualization. Hierarchical clustering was performed using Euclidean distance and complete linkage. Color intensity represents relative expression levels, with blue indicating lower values and red indicating higher values.

## Supplementary Tables

**Table S1. Proteomic data of 20 Healthy, 124 NATs, and 124 Tumor samples.** (**A**) Healthy1 dataset. (**B**) Healthy4 dataset.

**Table S2. Differential proteomic analysis of NAT and Tumor samples compared with Healthy samples.**

(**A**) Differentially expressed (DE) proteins in NAT vs. Healthy.

(**B**) DE proteins in Tumor vs. Healthy.

(**C**) Two-dimensional annotation enrichment analysis.

**Table S3. Proteomic subtypes of ESCC NATs.**

(**A**) NAT proteomic subtype.

(**B**) Clinicopathologic correlations.

(**C**) Differentially expressed proteins between NAT2 and NAT1.

(**D**) Upregulated pathways in NAT2.

(**E**) Downregulated pathways in NAT2.

(**F**) Differentially expressed phosphosites between NAT2 and NAT1.

(**G**) Cox regression analysis.

**Table S4. Estimated cell-type compositions in Healthy, NAT, and Tumor samples.**

**Table S5. Enrichment analysis of proteins classified by eight expression modes.**

(**A**) SU. (**B**) SD. (**C**) UU. (**D**) US. (**E**) UD. (**F**) DU. (**G**) DS. (**H**) DD.

**Table S6. Expression changes of known ESCC protein biomarkers across Healthy, NAT, and Tumor samples.**

**Table S7. Survival prediction analysis of ESCC patients.**

(**A**) Clinical characteristics of ESCC patients in training and test sets.

(**B**) Concordance indexes (C-indexes) of Ridge-Cox models for overall survival (OS) and disease-free survival (DFS).

(**C**, **D**) One-, three-, and five-year AUCs of Ridge-Cox models for OS (**C**) and DFS (**D**). (**E**–**G**) C-indexes of Lasso-Cox (**E**), sslasso (**F**), and glmboost (**G**) models for OS prediction.

**Table S8. Literature-supported validation of prognostic proteins identified in this study.**
